# Lifestyles, Food Consumption Frequencies, and Eating Behaviors among Three Main Disciplines of Undergraduate Students during the Early COVID-19 Outbreak in Thailand

**DOI:** 10.3390/nu15122765

**Published:** 2023-06-16

**Authors:** Yuraporn Sahasakul, Nantakan Amonsusawat, Phenphop Phansuea

**Affiliations:** Food and Nutrition Academic and Research Cluster, Institute of Nutrition, Mahidol University, Salaya, Phutthamonthon, Nakhon Pathom 73170, Thailand; yuraporn.sah@mahidol.edu (Y.S.); nantakan.amo@gmail.com (N.A.)

**Keywords:** lifestyles, food consumption frequencies, eating behaviors, disciplines of undergraduate students, COVID-19

## Abstract

University students’ lifestyles and dietary habits have been considerably impacted by the coronavirus disease 2019 (COVID-19) outbreak and its related policies and restrictions. An online cross-sectional survey was conducted from March to May 2020 to compare lifestyles, food consumption frequencies, and eating behaviors among three main disciplines of undergraduate students during the early COVID-19 outbreak in Thailand. The study involved 584 participants from Mahidol University, with 45.2% from Health Sciences (HS), 29.1% from Sciences and Technologies (ST), and 25.7% from Social Sciences and Humanities (SH). The results showed that ST students had the highest proportion of overweight and obese (33.5%) individuals, followed by HS (23.9%) and SH (19.3%) students. ST students skipped breakfast the most (34.7%), followed by SH (34%) and HS (30%) students. Furthermore, 60% of SH students spent 7 h or more daily on social media, and they had the least exercise and the highest frequency of ordering home-delivery food. SH students (43.3%) reported a higher likelihood of making unhealthier food choices and consuming fast food, processed meat, bubble tea, boxed fruit and vegetable juice, and crunchy snacks more frequently than students from other disciplines. The findings show that undergraduate students had poor eating behaviors and lifestyles during the early COVID-19 outbreak, highlighting the urgent need to promote food and nutrition security among students during and after the pandemic.

## 1. Introduction

Coronavirus disease 2019 (COVID-19) has significantly impacted the global population, with Thailand being no exception. This severe acute respiratory syndrome virus with potentially lethal effects was first reported in December 2019 in Wuhan City, Hubei Province, China. On 11 March 2020, the disease was identified as a novel coronavirus by the World Health Organization (WHO) [1]. The COVID-19 virus has rapidly spread worldwide, impacting many aspects of people’s daily lives [2]. It began as a significant public health crisis and later caused socioeconomic disturbances that had a broad impact on lifestyles and the supply of food and nutrition problems [3].

In order to prevent the spread, Thailand released the Outbreak Control Plan in March 2020. To monitor the coronavirus’s spread, the Ministry of Higher Education, Science, Research, and Innovation (MHESI) officially announced several measures [4,5]. As a result, universities were forced to temporarily close their campuses and switch from face-to-face lectures to online classes. Consequently, Mahidol University announced that all in-class teaching and activities should be suspended from March to May 2020 [6,7]. The preventive measures have led to many restrictions, which were found to be associated with eating behaviors and mental health in the international population [8,9].

Multiple studies indicate that transitioning to higher education can lead students to develop unhealthy routines [10,11,12]. The demanding nature of academic life often leads students to spend long hours studying without taking enough breaks. Consequently, students tend to consume caffeine and a diet high in sugar, fat, and sodium, while consuming fruits, vegetables, and whole grains falls below optimal levels [11,13]. Additionally, the lack of sufficient time available prevents students from engaging in sporting activities [11]. Poor lifestyles and food consumption habits can negatively impact the health of undergraduate students. A sedentary lifestyle and unhealthy eating habits can increase the risk of chronic diseases such as obesity, heart disease, and diabetes [14]. These conditions can weaken the immune system and make individuals more susceptible to infection and complications from COVID-19 [15,16,17]. Additionally, food supply disruptions and a lack of knowledge of healthy food access can negatively impact their health, leading to deficiencies in essential nutrients and vitamins, further weakening the immune system and increasing the risk of infection. A recent study revealed a significant weak negative correlation between poor diet quality and various health indicators, including higher BMI, poorer sleep quality, and higher stress levels among undergraduate students [18]. Furthermore, the study found a significant weak negative correlation between fruit and vegetable consumption and BMI, physical activity levels, and stress levels [18]. The pandemic significantly impacted the well-being and mental health of the general population, including university students [19,20]. Numerous studies have indicated increased anxiety, depression, stress, and fear during this period [19,20,21]. Higher perceived stress levels during the pandemic have been linked to increased motivation to seek out highly palatable and energy-dense foods, potentially contributing to obesity [22].

One of the key factors that can impact undergraduate students’ lifestyles and food consumption habits is their area of study. The core knowledge of different majors can impact students’ ability to access health knowledge and healthcare skills, thus affecting their eating behaviors and lifestyles [23]. In Thailand, we examine three main disciplines of study: Health Sciences (HS), Sciences and Technologies (ST), and Social Sciences and Humanities (SH). They represent a wide range of disciplines that may have different impacts on lifestyles and food consumption habits of students. For example, students in the HS discipline may have a more active lifestyle due to the nature of their coursework, while students in the ST and SH disciplines may have more sedentary lifestyles due to the emphasis on computer-based learning, lab work, and reading and writing tasks, respectively. Previous research at German University has shown that first-year female students enrolled in Natural Sciences, Mathematics, and Informatics had an increased risk of unhealthy lifestyles [24]. In addition, a study conducted during the COVID-19 pandemic collected data on food consumption and lifestyles among Thai undergraduate students [25]. This study enrolled a total of 464 participants, distributed across various disciplines: 45.7% in HS, 29.3% in ST, and 25.0% in SH. However, unlike our study, which analyzed data based on disciplines, the previous study analyzed the data for the entire student population. The study revealed that 76.1% of Thai undergraduate students spent 3–6 h per day engaged in online learning, 75.4% of students reported skipping breakfast at least three times per week, 63.8% of students reported sleeping 6–8 h per night, and 53.7% of students reported no exercise [25].

While the previous study provides valuable insights into the habits and behaviors of Thai undergraduate students during the pandemic, it is evident that the well-being of the undergraduate student population during the current epidemic is a matter of concern. As an exploratory study, our study aimed to extend this understanding by comparing the lifestyles, frequencies of food intake, and eating behaviors of Thai undergraduate students across three main disciplines of study during the early COVID-19 epidemic. By exploring the variations across different disciplines, we can better comprehend the specific challenges and opportunities for promoting healthier lifestyles and well-being among students. Promoting healthy food choices, regular physical activity, and nutrition awareness among college students is a priority to support their food and nutrition security and well-being during and after the epidemic.

## 2. Materials and Methods

### 2.1. Study Design and Ethics Approval

This cross-sectional descriptive study was conducted during the early COVID-19 period in Thailand between March and May 2020. A waiver of the requirement for written informed consent was granted. The study was approved by Mahidol University Central Institutional Review Board, Special Panel for COVID-19 (COA No. MU-COVID 2020.011/1307) and adhered to the tenets of the Declaration of Helsinki.

### 2.2. Sample Size Calculation

The sample size was determined using Taro Yamane’s method [26], with a 95% power of the test, based on the total undergraduate student population at Mahidol University in 2020. The determined sample size was 390 participants at a minimum.

### 2.3. Participants and Recruitment Procedures

**Figure 1:**
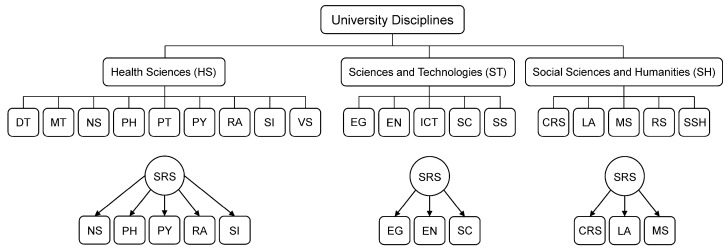
University disciplines. Abbreviations: HS—Health Sciences, ST—Sciences and Technologies, SH—Social Sciences and Humanities, DT—Faculty of Dentistry, MT—Faculty of Medical Technology, NS—Faculty of Nursing, PH—Faculty of Public Health, PT—Faculty of Physical Therapy, PY—Faculty of Pharmacy, RA—Faculty of Medicine Ramathibodi Hospital, SI—Faculty of Medicine Siriraj Hospital, VS—Faculty of Veterinary Science, EG—Faculty of Engineering, EN—Faculty of Environment and Resource Studies, ICT—Faculty of Information and Communication Technology, SC—Faculty of Science, SS—College of Sport Science, CRS—College of Religious Studies, LA—Faculty of Liberal Arts, MS—College of Music, RS—Ratchasuda College, SSH—Faculty of Social Sciences and Humanities, SRS—Simple Random Sampling.

Undergraduate students enrolled in different faculties across three campuses of Mahidol University: Salaya Campus (Nakhon Pathom), Phayathai Campus (Bangkok), and Bangkok Noi Campus (Bangkok), Thailand. The participants were invited to complete the electronic questionnaire via faculty/institutional websites, emails, Facebook pages, and Line groups’ networks. A stratified random sampling technique was used, and 11 faculties were selected independently from three disciplines using proportion allocation. An online-based platform (Google Forms) was used to distribute the e-questionnaire to undergraduate students through social networks. Representatives of disciplines from faculties and institutions were chosen randomly, as shown in Figure 1.

### 2.4. Research Instrument

A multi-element online self-administered questionnaire was designed using Google Forms documentation in Thai. The questionnaire underwent a review process involving five experts in relevant fields, including three nutritionists, one dietitian, and one statistician. Additionally, a pilot test was conducted with a sample of 30 undergraduate students, and slight modifications were made to enhance clarity and comprehensibility. The overall content validity index (CVI > 0.8) of the questionnaire was excellent. The questionnaire consisted of four sections: (1) sociodemographic characteristics (8 questions): academic year, gender, age, living arrangement, monthly allowance, change of allowance due to COVID-19 outbreak, height, and weight; (2) student lifestyles (7 questions): number of online subjects studied per week, hours per week of online studying, hours per week of self-study, hours per day of social media, screen time per day, minutes per week of exercise, and hours per night of sleep; and (3) frequencies of food intake (39 questions, 7 food categories): 3.1 rice and flour products, 3.2 meat products and nuts, beans, legumes, and their products, 3.3 vegetables and fruits, 3.4 high-fat food and fast food, 3.5 dried food and canned food, 3.6 beverages, and 3.7 snacks and desserts. To ensure the internal consistency of the food items included in the food frequency questionnaire (FFQ), Cronbach’s alpha was calculated specifically for this section. This assessment aimed to reduce the likelihood of false high internal consistency due to the impact of test length on the results. The obtained value (alpha = 0.81) indicated that the food items were strongly related. (4) Eating behaviors (6 questions): number of main meals consumed per day, meal skipping, type of main meals, snack consumption, mode of food access, and participants’ perception of the quality of food intake compared to the pre-COVID-19 period.

While it would be ideal for collecting data on various variables for both pre-pandemic and during the pandemic, we acknowledged the potential challenges posed by participant burden and the risk of incomplete responses due to a lengthy questionnaire. Therefore, to strike a balance between data comprehensiveness and participant engagement, we purposefully incorporated a question that allowed participants to self-evaluate the quality of their food intake during COVID-19 compared to the pre-pandemic period (the normal period). This approach aimed to ensure a manageable questionnaire length and minimize the potential for incomplete responses.

### 2.5. Statistical Analysis

SPSS version 27.0 (IBM Corp., Armonk, NY, USA) was used for all statistical analyses. The graph was generated with GraphPad Prism version 9.5.1 for Windows (GraphPad Software, San Diego, CA, USA). The Kolmogorov–Smirnov test was used to determine the normality of the data distribution. The means and standard deviations (SD) of continuous variables are presented. Counts and percentages were used to present categorical data. Chi-square tests (χ^2^) were used to determine the association between categorical variables. One-way ANOVA and Games–Howell post hoc analyses were used to assess differences between continuous variables. The Cramer’s V or the Partial eta-square for categorical or continuous variables were used for calculating effect size, respectively. The results were significant at *p* < 0.05.

## 3. Results

### 3.1. Sociodemographic Characteristics

Most participants (40.8%) were first-year students. Students in the HS discipline were the highest proportion (45.2%), followed by students in the ST discipline (29.1%) and students in the SH discipline (25.7%). It was found that 79.3% of the participants were women with a mean age of 19.6 ± 1.4 years. Most of them (84.4%) lived at home with their families. More than half of students (65.8%) reported having a monthly allowance of 7000 baht or less, and 69.9% reported a decreased allowance during the study period (Table 1).

**Figure 2:**
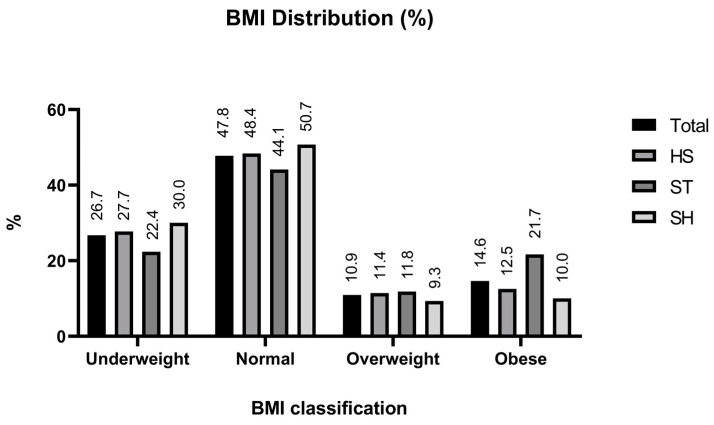
Comparison of percentages of BMI distribution among undergraduate students by disciplines of study during the early COVID-19 pandemic.

BMI was calculated based on the self-reported weight in kilograms divided by the self-reported height in meters squared and classified according to the Asia–Pacific guideline as shown in Table 1. Figure 2 shows a comparison of the BMI of students from different disciplines. ST students had the largest proportion of obese students (21.7%), followed by HS students (12.5%) and SH students (10%). While the order of the overweight group is reported to be the same, the highest proportion was found in the ST discipline (11.8%), followed closely by HS (11.4%) and SH (9.3%) disciplines, respectively.

### 3.2. Lifestyles

Table 2 displays information on student lifestyles during the early COVID-19 outbreak. According to the study, 69% of students had 1–5 online subjects per week, while 49% had 1–10 h of online study per week. There was a statistically significant association between the disciplines of study and the number of online subjects and a statistically significant relationship between the disciplines of study and the number of online studying hours (*p =* 0.002 and *p* < 0.001, respectively). Our results showed that ST students spent significantly more time studying online per week (14.1 ± 8.3 h) than HS (11.6 ± 6.7 h) and SH students (10.5 ± 6.6 h). Regarding self-study, HS students spent significantly more time on this per week (8.1 ± 6.7 h) than SH students (6.1 ± 5.7 h). Furthermore, the hours spent on social media and the disciplines of study were statistically significantly associated (*p =* 0.032). SH students used social media 8.7 ± 4.7 h daily, which was statistically significantly greater than ST and HS students at 7.3 ± 4.2 and 7.2 ± 3.9 h per day, respectively. The number of SH students who used online media for 7 h per day was the highest (60%) compared to ST (48.8%) and HS (47.0%) disciplines. In addition, most students across all disciplines spent 7 h or more daily on screens (76.9%). Regarding exercise and sleep duration, 54.5% of students exercised less than 150 min per week, and most slept 7 h or more per night (78.6%).

### 3.3. Frequencies of Food Intake

The frequencies of food intake during the COVID-19 pandemic among undergraduate students are presented in Table 3. It was found that for carbohydrate sources, white rice/polished rice noodles/white bread was still the most popular, with no difference among the three disciplines. For protein sources, fatty meat intake was associated with differences in disciplines of study, with 47.1% of ST, 32.9% of HS, and 32% of SH students consuming fatty meats almost every day, and approximately 12% of HS and ST students consuming once or more per day. The highest number of SH students (34.7%) consumed processed meat, such as bacon and sausages, almost daily. It was also found that SH students (8%) consumed processed meats more frequently than ST (7.1%) and HS (2.7%) discipline students on a daily basis. As for plant-based protein sources, our results showed that 7.3% of SH students were regular consumers of nuts, beans, legumes, and their products (consuming once or more per day), followed by students in ST (1.8%) and HS (1.5%), respectively. Additionally, 12.7% of students from the SH discipline reported low consumption of fresh vegetables, and 10% of the same discipline reported barely consuming fresh fruit (less than once a month). Almost half (47.4%) of students in the SH discipline, 34.7% of ST students, and 25.4% of HS students consumed fast food such as pizza, hamburgers, and French fries at least once weekly. Interestingly, 8% of the students in the SH discipline consumed bubble milk tea at least once a day. It was discovered that 17.3%, 11.1%, and 9.1% of students from the SH, ST, and HS disciplines drank bubble tea almost daily, respectively. Furthermore, students from SH (33.3%), ST (20%), and HS (19%) disciplines reported consuming crispy snacks such as potato chips nearly every day.

### 3.4. Eating Behaviors

Table 4 displays undergraduate students’ eating behaviors by disciplines of study. Early in the COVID-19 outbreak, it was reported that 44.2% of students ate three meals per day, 42.5% skipped meals, and the majority (76.6%) skipped breakfast. During confinement, 68% of students cooked their main meals, 26.7% relied on takeaway foods (cooked-to-order dishes/ready-made meals), and 5.3% relied on chilled/frozen foods. Interestingly, there was a statistically significant association between the types of main meals and the fields of study (*p =* 0.029). It was reported that 72.9% of ST students made their meals, followed by HS (69.3%) and SH students (60%). In addition to main meals, snacking was a common practice among the students (88.5%), with the afternoon being the most popular time for snacking (69.9%). It was also found that SH students ordered home-delivery food the most (32%), while the group who traveled to buy food by themselves the most was the HS discipline (78%), followed by ST (77.1%) and SH disciplines (64.7%), respectively. This study revealed that 43.3% of all students self-reported that their eating behaviors remained unchanged compared to the normal period. Among three disciplines, SH (43.3%), HS (35.2%), and ST (34.7%) students perceived that the pandemic led to an increase in unhealthy food choices. In contrast, ST (23.5%), HS (18.9%), and SH (16%) students reported consuming healthier items.

## 4. Discussion

### 4.1. Weight Status and BMI Classification among Undergraduate Students in Different Disciplines of Study during the Early COVID-19 Pandemic

Everyone was affected by the COVID-19 epidemic, and people had to adjust their lifestyles to cope with the pandemic, including universities, which operated as student learning centers. The teaching approach was modified to include online activities and online learning to encourage self-study. The transition from on-campus to online learning is linked to student behaviors. Several studies revealed that students who lack academic/social connection with their peers have reduced learning performance and, as a result, express various behavioral changes, including a decline in self-care practices [27,28]. Additionally, different disciplines have specific core knowledge, which could lead to different abilities to access health knowledge, and healthcare skills, thus affecting eating behaviors and lifestyles. In this study, students were classified as normal weight (47.8%), underweight (26.7%), obese (14.6%), and overweight (10.9%). According to the BMI categories, ST students had the highest proportion of overweight and obese individuals (33.5%), followed by HS students (23.9%) and SH students (19.3%), respectively.

Interestingly, a survey conducted among Thai undergraduate students in 2021 reported a higher prevalence of overweight and obese students (29.7%) and a lower prevalence of underweight individuals (23.1%) compared to our study [25]. While the proportions of students in each academic discipline (HS, ST, and SH) were similar in the two studies, the previous study did not analyze data separately for each study discipline. The discrepancy may be due to differences in sample characteristics such as gender, age, socioeconomic status, or timing and location of data collection. Overall, the current study highlights the importance of addressing BMI-related health issues among university students, and particularly ST students who appear to be at higher risk for overweight and obesity should be monitored closely (Supplementary Figure S1).

### 4.2. Lifestyles among Undergraduate Students in Different Disciplines of Study during the Early COVID-19 Pandemic

The COVID-19 pandemic transformed the teaching approach from traditional classroom learning to online learning [29]. This sudden change in teaching and learning styles resulted in many consequences for students’ learning modes and time spent daily. This study found that the HS and ST students allocated about 1.7–2.0 h per day to online studying, while ST and HS students allocated 1.1–1.2 h daily for self-study. On the other hand, SH students allocated less time to both online studying (1.5 h daily) and self-studying (0.9 h daily) than their peers. Interestingly, a previous study of Thai undergraduate students conducted in 2021 showed that 76.1% of students spent 3–6 h per day on online learning [25], whereas our study revealed that students spent less time studying online. One possible explanation for this difference is that our study was conducted in 2020 during the early COVID-19 outbreak when there was an abrupt transition from on-site to online studies, which may have posed challenges for both educators and students in adapting to the new normal of the online learning environment at that time.

Our study found that social media time spent was significantly associated with the fields of study. SH students spent significantly more time on social media than their peers, with the highest numbers of students using social media for 7 h or more daily. This might be because SH students spent fewer hours studying online and self-studying, which may allow them more time for other activities. Moreover, our findings revealed that most students in all fields had a high daily screen time, with an average of 10.4 ± 4.7 h per day. This is concerning given the association between excessive screen time and various health problems, including eye strain, headaches, neck and shoulder pain, disturbed sleep patterns, poorer mental health, overeating, unhealthy eating behaviors, and an increased risk of obesity and related diseases [30]. A recent systematic review concluded that using screens, particularly televisions, while eating can increase food intake due to exposure to unhealthy food marketing, a distraction from normal physiological eating cues, and emotional changes [31]. Due to the wide spread of on-demand videos, streaming services, and social media platforms, the concept of screen time has expanded beyond traditional television viewing and now includes a variety of electronic devices such as computers, tablets, and smartphones. Association of BMI and lifestyles (hours per week of online studying, hours per day of social media) among the three disciplines of study is shown in Supplementary Figure S2. Furthermore, previous research has shown the link between increased screen time and adverse health outcomes such as insomnia and being overweight, with 7 h as a critical threshold for screen time [32]. On the other hand, more physical activity and less screen time have been associated with better appetite control and eating habits [33]. Although there are no proposed restrictions on screen time for adolescents and adults, it is crucial to limit sedentary behaviors, particularly recreational screen time, to promote the health and well-being of students.

WHO recommends at least 150 min of moderate-intensity physical activity per week for people aged 18–67 years [34]. Even during normal circumstances, most students exercised less than the physical activity recommendation [35]. Previous studies conducted in China and Australia revealed reduced physical activity among youths during the outbreak [36,37]. Since the early outbreak of COVID-19, gatherings and outdoor activities have been restricted, which becomes an obstacle to exercise even more. Our study found that more than half of students had less than 150 min of physical activity per week, with 58%, 53.8%, and 52.4% of SH, HS, and ST students falling below this cutoff, respectively. This is consistent with the physical activity survey of Thai individuals aged 18–64 years in the early COVID-19 outbreak, which reported a decrease in the number of people engaging in at least 150 min of moderate physical activity per week compared to 2019 [38]. Additionally, a previous study during the COVID-19 pandemic demonstrated that 53.7% of Thai undergraduate students reported no exercise, while only 14.4% engaged in some form of exercise for at least 150 min per week [25]. Another study among German university students in 2019 demonstrated the amount of physical activity with the unit of the metabolic equivalent of task (MET)-min/week, showing that students in the Natural Sciences, Mathematics, and Informatics field (3427 MET-min/week) and students in the Language, Humanities and Cultural Studies field (3553 MET-min/week) reported the lowest physical activity, while students in the Education field (4312 MET-min/week), Medicine field (3981 MET-min/week), and Social Sciences, Media and Sports field (3844 MET-min/week) reported the highest physical activity levels [24]. Contrary to expectations, our study showed that although HS students had knowledge in health care, their adherence to exercise recommendations was not different from that of students in other fields. This unexpected finding may be attributed to the disruption of routine and limited access to exercise facilities during the early pandemic. The transition to online learning and implementing social distancing measures likely impacted students’ ability to engage in regular physical activities, leading to decreased exercise adherence across all disciplines.

Our study found that 78.6% of students slept 7 h or more per night, and obtaining at least 7 h of sleep is recommended. At the same time, our findings revealed that 23.5%, 23.3%, and 18.9% of students from the ST, SH, and HS disciplines slept less than 7 h per night, respectively. Inadequate sleep adversely affects health and cognitive processing [39]. Moreover, studies have shown that insufficient sleep can negatively impact eating behaviors and lead to overeating [40,41]. Strategies to promote healthy sleep habits, such as establishing regular sleep routines and avoiding electronic devices before bedtime, should be considered to improve university students’ overall health and well-being.

### 4.3. Eating Behaviors among Undergraduate Students in Different Disciplines of Study during the Early COVID-19 Pandemic

Breakfast is vital for students since it provides energy and influences their academic performance and daily activities. Many studies have demonstrated the importance of breakfast for academic performance [42,43]. Students who regularly ate breakfast had better academic performance than those who did not [44,45,46]. A previous study demonstrated that most HS students had breakfast [43], whereas a systematic review found that most university students did not eat breakfast for various reasons, such as a lack of time, food prices, and weight control [45]. Moreover, disease outbreaks and pandemics could cause transitory food insecurity, which may lead the individual to reduce the number of meals or amount, variety, and quality of meals [47]. Several studies found that skipping breakfast is also associated with unhealthy eating behaviors such as eating fewer fruits and vegetables [48,49], and high consumption of sugary drinks and snacks [48]. Additionally, some studies found that skipping breakfast is correlated with eating salty food and associated with prolonged sedentary activity [50]. A previous study revealed that 75.4% of Thai undergraduate students skipped breakfast thrice or more weekly [25]. Our study demonstrated that 32.5% of students skipped breakfast, and more than 70% of those who skipped meals in all disciplines were those who skipped breakfast. One-third of HS students did not consume breakfast even though they should know the advantages of breakfast due to their major in HS. Our result is consistent with a previous study in Bahrain reporting that more than half of HS students did not consume breakfast [51].

The present study found that 68% of students reported cooking their main meals during confinement. This finding contradicts a previous cross-sectional study conducted in Thailand during the non-lockdown period, which reported that 60.6% of Thai undergraduate students did not cook their meals [25]. It is important to note that these disparities between the two studies may be due to differences in time frames and data collection locations. Our study found that SH students consumed more ready-to-eat meals than other disciplines and had the highest home delivery rate. Cooking skills are reported to be related to eating behaviors; low cooking skills are associated with poor dietary behaviors such as low consumption of fresh vegetables and fruits, high consumption of ultra-processed food, the frequency of eating out, the frequency of takeaway and fast food meals, and high-energy food intake [52]. Based on our self-evaluation approach, 43.3% of all students self-reported that their eating behaviors remained unchanged compared to the normal period, which is higher than our initial expectation. Among three disciplines, SH (43.3%), HS (35.2%), and ST (34.7%) students perceived that the pandemic led to an increase in unhealthy food choices. In contrast, ST (23.5%), HS (18.9%), and SH (16.0%) students reported consuming healthier items. These results suggest that the pandemic has had mixed effects on students’ dietary habits, with some adopting healthier habits while others struggled to maintain healthy food choices.

### 4.4. Frequencies of Food Intake among Undergraduate Students in Different Disciplines of Study during the Early COVID-19 Pandemic

Animal fats are saturated fats and a risk factor for non-communicable diseases (NCDs) among working-age teenagers [53,54]. The highest percentage of students who ate meat with skin four times per week was ST students. Another type of meat high in fat and sodium is processed meat, which has various substances added during food processing. This research showed that students usually consumed processed meat in accordance with another report, which demonstrated that processed meat consumption is popular at this age [55]. This may be due to multi-culture eating habits, like Western diets. These often consist of red meat, processed meat, carbohydrate-rich foods, and refined rice groups, which are associated with the body’s inflammatory processes [56]. We also found an association between consumption frequencies and the fields of study. We found that 34.7% of SH students ate processed meat four or more times per week. In addition, SH students purchased frozen foods and Western food more often, with less home-cooking behavior than the other disciplines. However, the students who regularly ate legumes and nut products in the SH were twice as much as in the other two disciplines. Different types of nuts, beans, and legumes have different nutritional values [56]. Nuts, beans, and legumes are good snacks because they contain high protein, dietary fiber, vitamins, and minerals; however, some nuts are high in fat. Therefore, it is crucial to choose a suitable amount and preferably an unsalted type [57].

Our study found that a minority of students consumed vegetables and fruits at recommended levels. Specifically, when considering all types of vegetables (fresh, blanched/boiled, and stir-fried), only 44.7% of SH, 41.2% of ST, and 37.9% of HS students reported consuming vegetables at least once daily. Regarding fruit intake, less than half of the students consumed fresh fruits regularly, which falls short of the WHO recommendation [58]. Interestingly, HS students were more likely to consume fresh fruits regularly and were less likely to drink boxed fruit and vegetable juice than other disciplines. This suggests that HS students may have greater knowledge about healthy dietary habits. Fruits and vegetables are essential to a healthy diet, providing dietary fiber, vitamins, minerals, and phytochemicals, promoting good digestive and overall health. Students should be encouraged to increase their consumption of fresh fruits and vegetables in a whole form or as smoothies, which are more nutritious than boxed fruit and vegetable juices due to their lower sugar and higher fiber content.

Our study indicates that university students frequently consumed unhealthy diets during the early stages of the COVID-19 pandemic, as observed through both eating behaviors and FFQ. Supplementary Figure S3 shows the association of BMI, food consumption frequencies, and lifestyle (screen time per day) among the three disciplines of study. These poor dietary patterns indicate compromised diet quality and quantity, which have been linked with the severity of food and nutrition insecurity [47].

### 4.5. The Strengths and Limitations of This Study

This study has several strengths that should be acknowledged. This is the first cross-sectional study to examine eating behaviors, frequencies of food intake, and lifestyles during the early COVID-19 pandemic (March–May 2020) among undergraduate students across different disciplines. Additionally, the study was carefully designed with an appropriate sample size, sampling frame, and techniques, ensuring the reliability and validity of the findings. Moreover, providing an educational poster on healthy eating immediately after completing the questionnaire demonstrates a proactive approach to promoting healthy behaviors among participants. However, several limitations need to be considered. Firstly, the FFQ adopted in this study was originally designed for the Thai Population National Health Examination Survey, which included individuals aged 15 years and older. Our study population had a narrower age range, with a mean age of 19.6 years. Despite this discrepancy, the food groups and items identified in the FFQ are still relevant and appropriate for our study population. Secondly, due to the constraints imposed by the COVID-19 outbreak, the criterion validation of the FFQ was not conducted. As a result, our analysis was limited to assessing the frequency of food consumption rather than the estimation of nutrients, such as macronutrients and energy intake. Future research should consider utilizing a validated Semi-FFQ that enables the collection of comprehensive data on the frequency and quantity of food consumption, as well as macronutrients and energy intake. Thirdly, this study focused on a sample of undergraduate students from Mahidol University, and therefore, the findings may not be representative of the entire population of young adults in different circumstances. Caution should be exercised when generalizing the findings to broader populations. Fourthly, the self-reported nature of height and weight data in this online cross-sectional study introduces the possibility of discrepancy in BMI calculations. Fifth, while the COVID-19 pandemic began in 2019 and this survey was carried out between March and May 2020, shortly after the government announced work/study-from-home measures, the 2-month period may not fully capture the long-term effects of the pandemic on students’ lifestyles and behaviors. Lastly, the questionnaire did not include inquiries about the reasons for engaging in unhealthy behaviors during the COVID-19 pandemic or about nutrition knowledge and academic performance. Further studies should address these related questions in the questionnaire. Despite these limitations, the study provides data to guide future longitudinal work on these topics that has potential to improve university programs that promote students’ lifestyles, nutrition, and health on a university campus. Longitudinal studies should be conducted to investigate associations between students’ weight status, exercise adherence, and eating behaviors before, during, and after the pandemic. This will provide a more comprehensive understanding of the patterns and variables influencing their weight status and drive the development of focused approaches and strategies, during these difficult circumstances, to be on the cutting edge of healthy weight management.

## 5. Conclusions

The current study indicates that university students lived unhealthy lives during the first 2 months of the COVID-19 outbreak; most had unhealthy eating habits, poor fitness levels, and spent too much time on social media. University students may benefit from nutrition and health promotion programs to reduce the tendency to be overweight and obese, especially among ST students. The current study also found that SH students comprised the largest number of individuals who most frequently consumed unhealthy foods. Students’ activities during the COVID-19 pandemic showed that SH students had fewer online study hours than others, while their social media use hours were higher than the other two fields. However, the HS and ST students also had high social media hours at unhealthy levels. In further studies, the semi-FFQ, assessment of nutritional knowledge, and academic performance should be incorporated into the questionnaire to provide more comprehensive information. Course design and consulting services aimed at educating students and the empowerment needed to make effective choices about their health are a priority. Altogether, there is a high need to increase awareness and knowledge about healthy eating behaviors and lifestyles to promote food and nutrition security during and after the COVID-19 crisis. Information on balanced diets, healthy food choices, and proper lifestyles should be disseminated through social media, a communication channel widely used by students. Students can benefit greatly from the knowledge, adjust to the “new normal”, and develop sustainable habits for a healthier future.

## Figures and Tables

**Table 1:** Sociodemographic characteristics among undergraduate students by disciplines of study during the early COVID-19 pandemic.

Characteristics	Total (*n =* 584)	Disciplines of Study, *n* (%)		
		HS (*n =* 264)	ST (*n =* 170)	SH (*n =* 150)
Academic year				
1	238 (40.8)	92 (34.8)	70 (41.2)	76 (50.7)
2	184 (31.5)	101 (38.3)	42 (24.7)	41 (27.3)
3	85 (14.6)	41 (15.5)	24 (14.1)	20 (13.3)
≥4	77 (13.1)	30 (11.4)	34 (20.0)	13 (8.7)
Gender				
Female	463 (79.3)	234 (88.6)	115 (67.6)	114 (76.0)
Male	121 (20.7)	30 (11.4)	55 (32.4)	36 (24.0)
Age (years) (M ± SD)	19.6 ± 1.4	19.6 ± 1.3	19.6 ± 1.5	19.4 ± 1.4
Living arrangement				
Staying with family	493 (84.4)	227 (86.0)	140 (82.4)	126 (84.0)
Staying alone	91 (15.6)	37 (14.0)	30 (17.6)	24 (16.0)
Monthly allowance (baht)				
≤7000	384 (65.8)	174 (65.9)	114 (67.1)	96 (64.0)
7001–10,000	65 (11.1)	28 (10.6)	19 (11.2)	18 (12.0)
10,001–20,000	79 (13.5)	39 (14.8)	24 (14.1)	16 (10.7)
>20,000	56 (9.6)	23 (8.7)	13 (7.6)	20 (13.3)
Change of allowance due to the COVID-19 outbreak				
No change	176 (30.1)	76 (28.8)	53 (31.2)	47 (31.3)
Decrease	408 (69.9)	188 (71.2)	117 (68.8)	103 (68.7)
Anthropometric (M ± SD)				
Height (cm)	162.6 ± 0.7	161.2 ± 6.5	164.3 ± 8.6	163.2 ± 0.7
Body weight (kg)	56.9 ± 14.1	55.3 ± 11.9	59.8 ± 16.0	56.2 ± 15.1
BMI (kg/m^2^)	21.4 ± 4.6	21.2 ± 4.0	22.0 ± 5.1	21.0 ± 5.0
Underweight	156 (26.7)	73 (27.7)	38 (22.4)	45 (30.0)
Normal	279 (47.8)	128 (48.4)	75 (44.1)	76 (50.7)
Overweight	64 (10.9)	30 (11.4)	20 (11.8)	14 (9.3)
Obese	85 (14.6)	33 (12.5)	37 (21.7)	15 (10.0)

Data expressed as n (percentage) or the mean (SD) for categorical or continuous variables, respectively. Body mass index (BMI) classification: underweight (<18.5), normal weight (18.5–22.9), overweight (23.0–24.9), obese (≥25.0).

**Table 2:** Lifestyles among undergraduate students by disciplines of study during the early COVID-19 pandemic.

Characteristics	Total (*n =* 584)	χ^2^ or F	Disciplines of Study, *n* (%)			*p*	Effect Size
			HS (*n =* 264)	ST (*n =* 170)	SH (*n =* 150)		
Number of online subjects studied per week							
1–5	402 (69.0)	12.862	193 (73.1)	124 (72.9)	86 (57.3)	0.002 *	0.148
6–10	181 (31.0)		71 (26.9)	46 (27.1)	64 (42.7)		
Hours per week of online studying							
(M ± SD)	12.0 ± 7.3	11.041	11.6 ± 6.7 ^ab^	14.1 ± 8.3 ^abc^	10.5 ± 6.6 ^ac^	<0.001	0.037
1–10	286 (49.0)	9.554	130 (49.2)	60 (35.3)	96 (64.0)	<0.001 *	0.128
11–20	222 (38.0)		113 (42.8)	71 (41.8)	38 (25.3)		
21–30	76 (13.0)		21 (8.0)	39 (22.9)	16 (10.7)		
Hours per week of self-study							
(M ± SD)	7.4 ± 6.1	5.119	8.1 ± 6.7 ^a^	7.6 ± 5.5 ^b^	6.1 ± 5.7 ^ab^	0.006 *	0.017
1–10	511 (87.5)	4.213	223 (84.5)	152 (89.4)	136 (90.7)	0.378	0.085
11–20	40 (6.8)		22 (8.3)	10 (5.9)	8 (5.3)		
21–30	33 (5.7)		19 (7.2)	8 (4.7)	6 (4.0)		
Hours per day of social media							
(M ± SD)	7.6 ± 4.2	6.777	7.2 ± 3.9 ^a^	7.3 ± 4.2 ^b^	8.7 ± 4.7 ^ab^	0.001 *	0.023
<7	287 (49.1)	6.895	140 (53.0)	87 (51.2)	60 (40.0)	0.032 *	0.109
≥7	297 (50.9)		124 (47.0)	83 (48.8)	90 (60.0)		
Screen time per day							
(M ± SD)	10.4 ± 4.6	2.526	10.0 ± 4.2	10.4 ± 4.6	11.1 ± 5.2	0.081	0.009
<7	135 (23.1)	0.261	62 (23.5)	37 (21.8)	36 (24.0)	0.878	0.021
≥7	449 (76.9)		202 (76.5)	133 (78.2)	114 (76.0)		
Minutes per week of exercise							
(M ± SD)	173.3 ± 178.4	0.536	181.6 ± 191.3	168.4 ± 170.7	164.4 ± 163.4	0.585	0.002
<150	318 (54.5)	1.110	142 (53.8)	89 (52.4)	87 (58.0)	0.574	0.044
≥150	266 (45.5)		122 (46.2)	81 (47.6)	63 (42.0)		
Hours per night of sleep							
(M ± SD)	7.9 ± 2.1	1.028	7.9 ± 1.9	7.8 ± 2.1	8.1 ± 2.4	0.358	0.004
<7	125 (21.4)	1.742	50 (18.9)	40 (23.5)	35 (23.3)	0.419	0.055
≥7	459 (78.6)		214 (81.1)	130 (76.5)	115 (76.7)		

Data expressed as a count (percentage) or the mean (SD) for categorical or continuous variables, respectively. Pearson’s χ^2^ test or F test was used for categorical or continuous variables, respectively. * Significant at the *p*-value < 0.05; one-way ANOVA was used for comparisons among disciplines. ^abc^ Pair for *p* < 0.05 using the Bonferroni test for the difference within disciplines or Pearson’s χ^2^ test as appropriate. The Cramer’s V or the partial eta-square for categorical or continuous variables were used for calculating effect size, respectively.

**Table 3:** Frequencies of food intake among undergraduate students by disciplines of study during the early COVID-19 pandemic.

Food Items	Total (*n =* 584)	χ^2^	Disciplines of Study, *n* (%)			*p*	Effect Size
			HS (*n =* 264)	ST (*n =* 170)	SH (*n =* 150)		
1. Rice and flour products							
1.1 White rice/polished rice noodles/white bread							
<1 time/month	26 (4.4)	12.729	9 (3.4)	8 (4.7)	9 (6.0)	0.122	0.104
1–3 times/month	91 (15.6)		39 (14.8)	26 (15.3)	26 (17.4)		
1–3 times/week	137 (23.5)		72 (27.3)	30 (17.6)	35 (23.3)		
4–6 times/week	132 (22.6)		48 (18.2)	45 (26.5)	39 (26.0)		
≥1 time/day	198 (33.9)		96 (36.3)	61 (35.9)	41 (27.3)		
1.2 Brown rice/whole wheat bread/cereal bread							
<1 time/month	127 (21.7)	6.690	60 (22.7)	38 (22.4)	29 (19.3)	0.570	0.076
1–3 times/month	173 (29.7)		78 (29.6)	44 (25.9)	51 (34.0)		
1–3 times/week	141 (24.1)		70 (26.5)	42 (24.7)	29 (19.3)		
4–6 times/week	76 (13.0)		31 (11.7)	24 (14.1)	21 (14.0)		
≥1 time/day	67 (11.5)		25 (9.5)	22 (12.9)	20 (13.4)		
1.3 Sticky rice							
<1 time/month	105 (18.0)	5.116	52 (19.7)	28 (16.5)	25 (16.7)	0.745	0.066
1–3 times/month	264 (45.2)		113 (42.8)	79 (46.5)	72 (48.0)		
1–3 times/week	152 (26.0)		65 (24.6)	49 (28.8)	38 (25.3)		
4–6 times/week	38 (6.5)		21 (8.0)	7 (4.1)	10 (6.7)		
≥1 time/day	25 (4.3)		13 (4.9)	7 (4.1)	5 (3.3)		
2. Meat products and nuts, beans, legumes, and their products							
2.1 Fatty meats/meats with skin							
<1 time/month	41 (7.0)	17.174	18 (6.8)	7 (4.1)	16 (10.7)	0.028 *	0.121
1–3 times/month	124 (21.2)		62 (23.5)	32 (18.8)	30 (20.0)		
1–3 times/week	204 (34.9)		97 (36.8)	51 (30.0)	56 (37.3)		
4–6 times/week	151 (25.9)		56 (21.2)	60 (35.3)	35 (23.3)		
≥1 time/day	64 (11.0)		31 (11.7)	20 (11.8)	13 (8.7)		
2.2 Meats without skin and lean meats							
<1 time/month	25 (4.3)	8.044	11 (4.1)	6 (3.5)	8 (5.3)	0.429	0.083
1–3 times/month	85 (14.5)		39 (14.8)	18 (10.6)	28 (18.7)		
1–3 times/week	195 (33.4)		91 (34.5)	54 (31.8)	50 (33.3)		
4–6 times/week	188 (32.2)		80 (30.3)	66 (38.8)	42 (28.0)		
≥1 time/day	91 (15.6)		43 (16.3)	26 (15.3)	22 (14.7)		
2.3 Boiled egg/poached egg/fried egg/omelet							
<1 time/month	13 (2.2)	13.230	6 (2.3)	5 (2.9)	2 (1.3)	0.104	0.106
1–3 times/month	51 (8.7)		23 (8.7)	9 (5.3)	19 (12.7)		
1–3 times/week	169 (28.9)		86 (32.6)	44 (25.9)	39 (26.0)		
4–6 times/week	235 (40.3)		105 (39.8)	77 (45.3)	53 (35.3)		
≥1 time/day	116 (19.9)		44 (16.6)	35 (20.6)	37 (24.7)		
2.4 Fish (excluding sea fish)							
<1 time/month	43 (7.4)	14.222	19 (7.2)	15 (8.8)	9 (6.0)	0.076	0.110
1–3 times/month	165 (28.2)		74 (28.0)	50 (29.4)	41 (27.3)		
1–3 times/week	210 (36.0)		97 (36.7)	52 (30.6)	61 (40.7)		
4–6 times/week	125 (21.4)		58 (22.0)	45 (26.5)	22 (14.7)		
≥1 time/day	41 (7.0)		16 (6.1)	8 (4.7)	17 (11.3)		
2.5 Seafoods							
<1 time/month	86 (14.7)	8.228	34 (12.9)	31 (18.2)	21 (14.0)	0.412	0.084
1–3 times/month	225 (38.5)		111 (42.0)	55 (32.4)	59 (39.3)		
1–3 times/week	181 (31.0)		83 (31.4)	56 (32.9)	42 (28.0)		
4–6 times/week	77 (13.2)		30 (11.4)	25 (14.7)	22 (14.7)		
≥1 time/day	15 (2.6)		6 (2.3)	3 (1.8)	6 (4.0)		
2.6 Animal blood/internal organs of animals							
<1 time/month	269 (46.0)	9.220	118 (44.7)	81 (47.7)	70 (46.7)	0.324	0.089
1–3 times/month	203 (34.8)		96 (36.3)	59 (34.7)	48 (32.0)		
1–3 times/week	84 (14.4)		43 (16.3)	22 (12.9)	19 (12.7)		
≥4 times/week	28 (4.8)		7 (2.7)	8 (4.7)	13 (8.6)		
2.7 Processed meats							
<1 time/month	50 (8.6)	23.967	27 (10.2)	12 (7.1)	11 (7.3)	0.002 *	0.143
1–3 times/month	168 (28.8)		95 (36.0)	45 (26.4)	28 (18.7)		
1–3 times/week	215 (36.8)		91 (34.5)	65 (38.2)	59 (39.3)		
4–6 times/week	120 (20.5)		44 (16.6)	36 (21.2)	40 (26.7)		
≥1 time/day	31 (5.3)		7 (2.7)	12 (7.1)	12 (8.0)		
2.8 Nuts, beans, legumes, and their products (excluding beverages)							
<1 time/month	139 (23.8)	24.511	63 (23.9)	47 (27.6)	29 (19.3)	0.002 *	0.145
1–3 times/month	219 (37.5)		116 (43.9)	50 (29.5)	53 (35.4)		
1–3 times/week	164 (28.1)		64 (24.2)	57 (33.5)	43 (28.7)		
4–6 times/week	44 (7.5)		17 (6.5)	13 (7.6)	14 (9.3)		
≥1 time/day	18 (3.1)		4 (1.5)	3 (1.8)	11 (7.3)		
3. Vegetables and fruits							
3.1 Fresh vegetables							
<1 time/month	61 (10.4)	9.690	23 (8.7)	19 (11.2)	19 (12.7)	0.287	0.091
1–3 times/month	107 (18.4)		43 (16.3)	34 (20.0)	30 (20.0)		
1–3 times/week	157 (26.9)		82 (31.1)	36 (21.2)	39 (26.0)		
4–6 times/week	155 (26.5)		67 (25.3)	54 (31.7)	34 (22.6)		
≥1 time/day	104 (17.8)		49 (18.6)	27 (15.9)	28 (18.7)		
3.2 Blanched vegetables/boiled vegetables							
<1 time/month	79 (13.5)	8.780	27 (10.2)	27 (15.9)	25 (16.7)	0.361	0.087
1–3 times/month	125 (21.4)		61 (23.1)	32 (18.8)	32 (21.3)		
1–3 times/week	154 (26.4)		72 (27.3)	41 (24.1)	41 (27.3)		
4–6 times/week	146 (25.0)		72 (27.3)	45 (26.5)	29 (19.4)		
≥1 time/day	80 (13.7)		32 (12.1)	25 (14.7)	23 (15.3)		
3.3 Stir-fried vegetables							
<1 time/month	69 (11.8)	8.125	31 (11.7)	22 (12.9)	16 (10.7)	0.421	0.083
1–3 times/month	130 (22.3)		56 (21.2)	34 (20.0)	40 (26.6)		
1–3 times/week	196 (33.5)		101 (38.3)	52 (30.6)	43 (28.7)		
4–6 times/week	136 (23.3)		57 (21.6)	44 (25.9)	35 (23.3)		
≥1 time/day	53 (9.1)		19 (7.2)	18 (10.6)	16 (10.7)		
3.4 Fresh fruits							
<1 time/month	32 (5.5)	17.915	5 (1.9)	12 (7.1)	15 (10.0)	0.022 *	0.124
1–3 times/month	93 (15.9)		40 (15.1)	30 (17.6)	23 (15.3)		
1–3 times/week	182 (31.2)		87 (33.0)	51 (30.0)	44 (29.4)		
4–6 times/week	188 (32.2)		97 (36.7)	49 (28.8)	42 (28.0)		
≥1 time/day	89 (15.2)		35 (13.3)	28 (16.5)	26 (17.3)		
3.5 Processed fruits							
<1 time/month	313 (53.6)	15.961	148 (56.1)	96 (56.5)	69 (46.0)	0.043 *	0.117
1–3 times/month	178 (30.5)		76 (28.8)	51 (30.0)	51 (34.0)		
1–3 times/week	60 (10.2)		26 (9.8)	15 (8.8)	19 (12.7)		
≥4 times/week	33 (5.7)		14 (5.3)	8 (4.7)	11 (7.3)		
4. High-fat food and fast food							
4.1 Curry with coconut milk/oil							
<1 time/month	112 (19.2)	15.522	48 (18.2)	31 (18.2)	33 (22.0)	0.050	0.115
1–3 times/month	172 (29.5)		94 (35.6)	49 (28.8)	29 (19.3)		
1–3 times/week	186 (31.8)		75 (28.4)	60 (35.3)	51 (34.0)		
4–6 times/week	101 (17.3)		43 (16.3)	27 (15.9)	31 (20.7)		
≥1 time/day	13 (2.2)		4 (1.5)	3 (1.8)	6 (4.0)		
4.2 Western fast food							
<1 time/month	120 (20.5)	26.443	63 (23.9)	39 (22.9)	18 (12.0)	0.001 *	0.150
1–3 times/month	267 (45.8)		134 (50.7)	72 (42.4)	61 (40.6)		
1–3 times/week	141 (24.1)		53 (20.1)	40 (23.5)	48 (32.0)		
4–6 times/week	45 (7.7)		11 (4.2)	15 (8.8)	19 (12.7)		
≥1 time/day	11 (1.9)		3 (1.1)	4 (2.4)	4 (2.7)		
4.3 Fried foods							
<1 time/month	35 (6.0)	6.519	15 (5.7)	13 (7.6)	7 (4.7)	0.589	0.075
1–3 times/month	139 (23.8)		61 (23.1)	44 (25.9)	34 (22.7)		
1–3 times/week	230 (39.4)		112 (42.4)	61 (35.9)	57 (38.0)		
4–6 times/week	152 (26.0)		61 (23.1)	44 (25.9)	47 (31.3)		
≥1 time/day	28 (4.8)		15 (5.7)	8 (4.7)	5 (3.3)		
4.4 Grilled foods							
<1 time/month	93 (15.9)	9.748	40 (15.1)	32 (18.8)	21 (14.0)	0.283	0.091
1–3 times/month	244 (41.8)		124 (47.0)	66 (38.8)	54 (36.0)		
1–3 times/week	148 (25.3)		62 (23.5)	43 (25.3)	43 (28.7)		
4–6 times/week	86 (14.8)		32 (12.1)	27 (15.9)	27 (18.0)		
≥1 time/day	13 (2.2)		6 (2.3)	2 (1.2)	5 (3.3)		
5. Dried food and canned food							
5.1 Instant noodles							
<1 time/month	92 (15.8)	12.417	41 (15.5)	30 (17.6)	21 (14.0)	0.134	0.103
1–3 times/month	180 (30.8)		85 (32.2)	53 (31.2)	42 (28.0)		
1–3 times/week	183 (31.3)		92 (34.9)	48 (28.3)	43 (28.7)		
4–6 times/week	96 (16.4)		36 (13.6)	31 (18.2)	29 (19.3)		
≥1 time/day	33 (5.7)		10 (3.8)	8 (4.7)	15 (10.0)		
5.2 Canned fish							
<1 time/month	245 (42.0)	14.264	111 (42.0)	75 (44.1)	59 (39.3)	0.075	0.111
1–3 times/month	212 (36.3)		103 (39.0)	63 (37.1)	46 (30.7)		
1–3 times/week	88 (15.0)		39 (14.8)	20 (11.7)	29 (19.3)		
≥4 times/week	39 (6.7)		11 (4.2)	12 (7.1)	16 (10.7)		
5.3 Chili paste							
<1 time/month	161 (27.6)	6.456	77 (29.2)	49 (28.8)	35 (23.3)	0.596	0.074
1–3 times/month	152 (26.0)		62 (23.5)	47 (27.7)	43 (28.7)		
1–3 times/week	154 (26.4)		68 (25.7)	44 (25.9)	42 (28.0)		
4–6 times/week	86 (14.7)		42 (15.9)	25 (14.7)	19 (12.7)		
≥1 time/day	31 (5.3)		15 (5.7)	5 (2.9)	11 (7.3)		
5.4 Salted meats							
<1 time/month	233 (39.9)	11.808	107 (40.5)	73 (42.9)	53 (35.3)	0.160	0.101
1–3 times/month	219 (37.5)		108 (40.9)	57 (33.5)	54 (36.0)		
1–3 times/week	100 (17.1)		42 (15.9)	29 (17.1)	29 (19.4)		
4–6 times/week	27 (4.6)		6 (2.3)	9 (5.3)	12 (8.0)		
≥1 time/day	5 (0.9)		1 (0.4)	2 (1.2)	2 (1.3)		
6. Beverages							
6.1 Soft drinks/flavored syrup							
<1 time/month	130 (22.3)	11.426	59 (22.3)	42 (24.7)	29 (19.3)	0.179	0.099
1–3 times/month	156 (26.7)		80 (30.3)	42 (24.7)	34 (22.7)		
1–3 times/week	161 (27.6)		75 (28.4)	47 (27.6)	39 (26.0)		
4–6 times/week	97 (16.6)		34 (12.9)	27 (15.9)	36 (24.0)		
≥1 time/day	40 (6.8)		16 (6.1)	12 (7.1)	12 (8.0)		
6.2 Coffee/tea							
<1 time/month	149 (25.5)	10.609	71 (26.9)	42 (24.7)	36 (24.0)	0.225	0.095
1–3 times/month	146 (25.0)		72 (27.3)	39 (22.9)	35 (23.3)		
1–3 times/week	139 (23.8)		65 (24.6)	42 (24.7)	32 (21.3)		
4–6 times/week	95 (16.3)		41 (15.5)	29 (17.1)	25 (16.7)		
≥1 time/day	55 (9.4)		15 (5.7)	18 (10.6)	22 (14.7)		
6.3 Bubble milk tea							
<1 time/month	185 (31.7)	17.374	86 (32.6)	63 (37.1)	36 (24.1)	0.026 *	0.122
1–3 times/month	184 (31.5)		8 (31.8)	50 (29.4)	50 (33.3)		
1–3 times/week	146 (25.0)		70 (26.5)	38 (22.4)	38 (25.3)		
4–6 times/week	48 (8.2)		20 (7.6)	14 (8.2)	14 (9.3)		
≥1 time/day	21 (3.6)		4 (1.5)	5 (2.9)	12 (8.0)		
6.4 Energy drink/sports drink							
<1 time/month	463 (79.3)	20.370	222 (84.1)	136 (80.0)	105 (70.0)	0.002 *	0.132
1–3 times/month	84 (14.4)		34 (12.9)	24 (14.1)	26 (17.3)		
1–3 times/week	23 (3.9)		7 (2.6)	6 (3.5)	10 (6.7)		
≥4 times/week	14 (2.4)		1 (0.4)	4 (2.4)	9 (6.0)		
6.5 Boxed fruit juice/boxed vegetable juice							
<1 time/month	256 (43.8)	18.563	125 (47.3)	72 (42.4)	59 (39.4)	0.017 *	0.126
1–3 times/month	172 (29.5)		83 (31.4)	54 (31.8)	35 (23.3)		
1–3 times/week	100 (17.1)		40 (15.2)	25 (14.7)	35 (23.3)		
4–6 times/week	43 (7.4)		15 (5.7)	13 (7.6)	15 (10.0)		
≥1 time/day	13 (2.2)		1 (0.4)	6 (3.5)	6 (4.0)		
6.6 Fresh vegetable/fruit smoothies							
<1 time/month	245 (42.0)	7.368	110 (41.6)	78 (45.9)	57 (38.0)	0.498	0.079
1–3 times/month	177 (30.3)		89 (33.7)	40 (23.5)	48 (32.0)		
1–3 times/week	29 (18.6)		43 (16.3)	37 (21.8)	29 (19.3)		
4–6 times/week	10 (6.0)		15 (5.7)	10 (5.9)	10 (6.7)		
≥1 time/day	6 (3.1)		7 (2.7)	5 (2.9)	6 (4.0)		
6.7 Fresh milk/sweet milk/flavored milk							
<1 time/month	125 (21.4)	21.336	55 (20.8)	41 (24.1)	29 (19.3)	0.006 *	0.135
1–3 times/month	148 (25.3)		86 (32.6)	37 (21.8)	25 (16.7)		
1–3 times/week	147 (25.2)		66 (25.0)	42 (24.7)	39 (26.0)		
4–6 times/week	102 (17.5)		37 (14.0)	30 (17.6)	35 (23.3)		
≥1 time/day	62 (10.6)		20 (7.6)	20 (11.8)	22 (14.7)		
6.8 Low-fat milk/skimmed milk							
<1 time/month	178 (30.5)	14.939	77 (29.2)	61 (35.9)	40 (26.7)	0.060	0.113
1–3 times/month	167 (28.6)		77 (29.2)	46 (27.0)	44 (29.3)		
1–3 times/week	125 (21.4)		64 (24.2)	36 (21.2)	25 (16.7)		
4–6 times/week	67 (11.5)		29 (11.0)	18 (10.6)	20 (13.3)		
≥1 time/day	47 (8.0)		17 (6.4)	9 (5.3)	21 (14.0)		
6.9 Natural yoghurt/flavored yoghurt/drinking yoghurt							
<1 time/month	149 (25.5)	6.652	71 (26.9)	45 (26.5)	33 (22.0)	0.575	0.075
1–3 times/month	188 (32.2)		94 (35.6)	51 (30.0)	43 (28.7)		
1–3 times/week	139 (23.8)		58 (22.0)	40 (23.5)	41 (27.3)		
4–6 times/week	76 (13.0)		28 (10.6)	25 (14.7)	23 (15.3)		
≥1 time/day	32 (5.5)		13 (4.9)	9 (5.3)	10 (6.7)		
6.10 Soy milk/nut milk							
<1 time/month	169 (28.9)	9.657	75 (28.4)	52 (30.6)	42 (28.0)	0.290	0.091
1–3 times/month	174 (29.8)		85 (32.2)	52 (30.6)	37 (24.7)		
1–3 times/week	144 (24.7)		66 (25.0)	42 (24.7)	36 (24.0)		
4–6 times/week	66 (11.3)		28 (10.6)	17 (10.0)	21 (14.0)		
≥1 time/day	31 (5.3)		10 (3.8)	7 (4.1)	14 (9.3)		
7. Snacks and desserts							
7.1 Bakeries							
<1 time/month	78 (13.4)	13.152	38 (14.4)	24 (14.1)	16 (10.7)	0.100	0.107
1–3 times/month	189 (32.3)		97 (36.7)	49 (28.8)	43 (28.6)		
1–3 times/week	202 (34.6)		88 (33.3)	63 (37.1)	51 (34.0)		
4–6 times/week	90 (15.4)		34 (12.9)	28 (16.5)	28 (18.7)		
≥1 time/day	25 (4.3)		7 (2.7)	6 (3.5)	12 (8.0)		
7.2 Thai sugar- and egg-rich desserts							
<1 time/month	268 (45.9)	23.366	118 (4.7)	79 (46.5)	71 (47.3)	0.001 *	0.141
1–3 times/month	201 (34.4)		108 (40.9)	54 (31.7)	39 (26.0)		
1–3 times/week	92 (15.8)		36 (13.6)	27 (15.9)	29 (19.4)		
≥4 times/week	23 (3.9)		2 (0.8)	10 (5.9)	11 (7.3)		
7.3 Thai coconut-based desserts							
<1 time/month	234 (40.1)	18.747	102 (38.6)	76 (44.7)	56 (37.3)	0.005 *	0.127
1–3 times/month	216 (37.0)		108 (40.9)	61 (35.9)	47 (31.4)		
1–3 times/week	101 (17.3)		45 (17.1)	27 (15.9)	29 (19.3)		
≥4 times/week	33 (5.6)		9 (3.4)	6 (3.5)	18 (12)		
7.4 Thai syrup-based desserts							
<1 time/month	254 (43.5)	16.793	113 (42.8)	81 (47.7)	60 (40.0)	0.032 *	0.120
1–3 times/month	213 (36.5)		108 (40.9)	57 (33.5)	48 (32.0)		
1–3 times/week	93 (15.9)		38 (14.4)	26 (15.3)	29 (19.3)		
≥4 times/week	24 (4.1)		5 (1.9)	6 (3.5)	13 (8.7)		
7.5 Crispy snacks							
<1 time/month	73 (12.5)	18.580	31 (11.7)	24 (14.1)	18 (12.0)	0.017 *	0.126
1–3 times/month	184 (31.5)		98 (37.1)	55 (32.4)	31 (20.7)		
1–3 times/week	193 (33.0)		85 (32.2)	57 (33.5)	51 (34.0)		
4–6 times/week	95 (16.3)		35 (13.3)	25 (14.7)	35 (23.3)		
≥1 time/day	39 (6.7)		15 (5.7)	9 (5.3)	15 (10.0)		

Data expressed as a count (percentage). * Significant at the *p*-value < 0.05; Pearson’s χ^2^ test was used for comparisons among disciplines. The Cramer’s V was used for calculating effect size.

**Table 4:** Eating behaviors among undergraduate students by disciplines of study during the early COVID-19 pandemic.

Eating Behaviors	Total (*n =* 584)	χ^2^	Disciplines of Study, *n* (%)			*p*	Effect Size
			HS (*n =* 264)	ST (*n =* 170)	SH (*n =* 150)		
Main meal per day							
<3 meals	326 (55.8)	2.525	138 (52.3)	101 (59.4)	87 (58.0)	0.283	0.066
3 meals	258 (44.2)		126 (47.7)	69 (40.6)	63 (42.0)		
Meal skipping							
No skipping	336 (57.5)	2.737	154 (58.3)	94 (55.3)	88 (58.7)	0.841	0.048
Skipping breakfast	190 (32.5)		80 (30.0)	59 (34.7)	51 (34.0)		
Skipping lunch	28 (4.9)		14 (5.3)	9 (5.3)	5 (3.3)		
Skipping dinner	30 (5.1)		16 (6.1)	8 (4.7)	6 (4.0)		
Type of main meal							
Owned cooked dishes	397 (68.0)	10.763	183 (69.3)	124 (72.9)	90 (60.0)	0.029 *	0.096
Takeaway foods	156 (26.7)		68 (25.8)	42 (24.7)	46 (30.7)		
Chilled/frozen foods	31 (5.3)		13 (4.9)	4 (2.4)	14 (9.3)		
Snack consumption ^a^	517 (88.5)	0.999	236 (89.4)	147 (86.5)	134 (89.3)	0.607	0.041
Before breakfast	43 (7.4)	1.167	18 (6.8)	11 (6.5)	14 (9.3)	0.558	0.045
Brunch	202 (34.6)	0.268	89 (33.7)	62 (36.5)	51 (34.0)	0.875	0.021
Afternoon	408 (69.9)	2.560	189 (71.6)	111 (65.3)	108 (72.0)	0.278	0.066
Before bedtime	98 (16.8)	2.824	37 (14.0)	32 (18.8)	29 (19.3)	0.244	0.070
Mode of food access							
Self-purchasing	434 (74.3)	14.682	206 (78.0)	131 (77.1)	97 (64.7)	0.005 *	0.112
Home delivery	124 (21.2)		44 (16.7)	32 (18.8)	48 (32.0)		
Domestic sources	26 (4.5)		14 (5.3)	7 (4.1)	5 (3.3)		
Self-perception toward the quality of food intake							
No change	253 (43.3)	5.201	121 (45.9)	71 (41.8)	61 (40.7)	0.267	0.067
Eating unhealthier	217 (37.2)		93 (35.2)	59 (34.7)	65 (43.3)		
Eating healthier	114 (19.5)		50 (18.9)	40 (23.5)	24 (16.0)		

* Chi-square test *p* < 0.05; values are expressed as mean and standard deviation (M ± SD) for continuous variables or as number and percentage (*n* (%)) for categorical variables. The Cramer’s V was used for calculating effect size. ^a^ Multiple answers.

## Data Availability

Data associated with the paper are available upon request.

## Supplementary Materials

The following are available online at https://www.mdpi.com/article/10.3390/nu15122765/s1, Figure S1: BMI Distribution among Undergraduate Students by Disciplines of Study, Figure S2: Association of BMI and Lifestyles (Hours per Week of Online Studying, Hours per Day of Social Media) among the Three Disciplines of Study, Figure S3: Association of BMI, Food Consumption Frequencies, and Lifestyle (Screen Time per Day) among the Three Disciplines of Study.

## References

[1] Zhu N., Zhang D., Wang W., Li X., Yang B., Song J., Zhao X., Huang B., Shi W., Lu R. (2020). A Novel Coronavirus from Patients with Pneumonia in China, 2019. N. Engl. J. Med..

[2] World Health Organization Novel Coronavirus (2019-nCoV) Situation Report 21st January. https://www.who.int/docs/default-source/coronaviruse/situation-reports/20200121-sitrep-1-2019-ncov.pdf?sfvrsn=20a99c10_4.

[3] United Nations (2020). Policy Brief: The Impact of COVID-19 on Food Security and Nutrition in 2020 June.

[4] Ministry of Public Health (Thailand) (2020). 3rd Announcement: Name and Major Symptoms of Dangerous Communicable Diseases.

[5] Emergency Operation Center (2020). Department of Disease Control. COVID-19 Situation Reports.

[6] Mahidol University (2020). Announcement of the President of Mahidol University on Suspension of All Classroom Teaching and Learning in Mahidol University and Work from Home Guideline for Mahidol University Staff Due to the Outbreak of Coronavirus 2019 (COVID-19).

[7] Mahidol University (2020). Announcement of Mahidol University Guidelines for Teaching and Learning, Work from Home and Social Distancing Measures to Respond to the Coronavirus 2019 (COVID-19) Outbreak.

[8] Ammar A., Brach M., Trabelsi K., Chtourou H., Boukhris O., Masmoudi L., Bouaziz B., Bentlage E., How D., Ahmed M. (2020). Effects of COVID-19 home confinement on eating behaviour and physical activity: Results of the ECLB-COVID19 international online survey. Nutrients.

[9] Ammar A., Trabelsi K., Brach M., Chtourou H., Boukhris O., Masmoudi L., Bouaziz B., Bentlage E., How D., Ahmed M. (2020). Effects of home confinement on mental health and lifestyle behaviours during the COVID-19 outbreak: Insight from the “ECLB-COVID19” multi countries survey. medRxiv.

[10] Deliens T., Deforche B., De Bourdeaudhuij I., Clarys P. (2015). Determinants of physical activity and sedentary behaviour in university students: A qualitative study using focus group discussions. BMC Public Health.

[11] Aceijas C., Waldhäusl S., Lambert N., Cassar S., Bello-Corassa R. (2017). Determinants of health-related lifestyles among university students. Perspect. Public Health.

[12] Maillet M.A., Grouzet F.M.E. (2023). Understanding changes in eating behavior during the transition to university from a self-determination theory perspective: A systematic review. J. Am. Coll. Health.

[13] Strong K.A., Parks S.L., Anderson E., Winett R., Davy B.M. (2008). Weight gain prevention: Identifying theory-based targets for health behavior change in young adults. J. Am. Diet Assoc..

[14] Arocha Rodulfo J.I. (2019). Sedentary lifestyle a disease from xxi century. Clin. Investig. Arter..

[15] Azarpazhooh M.R., Morovatdar N., Avan A., Phan T.G., Divani A.A., Yassi N., Stranges S., Silver B., Biller J., Tokazebani Belasi M. (2020). COVID-19 Pandemic and Burden of Non-Communicable Diseases: An Ecological Study on Data of 185 Countries. J. Stroke Cereb. Dis..

[16] Alamri E.S. (2021). Effects of COVID-19 home confinement on eating behavior: A review. J. Public Health Res..

[17] Cava E., Neri B., Carbonelli M.G., Riso S., Carbone S. (2021). Obesity pandemic during COVID-19 outbreak: Narrative review and future considerations. Clin. Nutr..

[18] Doak S., Kearney J.M., McCormack J.M., Keaver L. (2023). The relationship between diet and lifestyle behaviours in a sample of higher education students; a cross-sectional study. Clin. Nutr. ESPEN.

[19] Wang C., Pan R., Wan X., Tan Y., Xu L., Ho C.S., Ho R.C. (2020). Immediate Psychological Responses and Associated Factors during the Initial Stage of the 2019 Coronavirus Disease (COVID-19) Epidemic among the General Population in China. Int. J. Environ. Res. Public Health.

[20] Machado B.C., Moreira C.S., Correia M., Veiga E., Gonçalves S. (2023). Coping as a Mediator and Moderator between Psychological Distress and Disordered Eating Behaviors and Weight Changes during the COVID-19 Pandemic. Int. J. Environ. Res. Public Health.

[21] Serafini G., Parmigiani B., Amerio A., Aguglia A., Sher L., Amore M. (2020). The psychological impact of COVID-19 on the mental health in the general population. Qjm.

[22] Smith K.R., Jansen E., Thapaliya G., Aghababian A.H., Chen L., Sadler J.R., Carnell S. (2021). The influence of COVID-19-related stress on food motivation. Appetite.

[23] Rueda-Medina B., Gomez-Urquiza J.L., Tapia-Haro R., Casas-Barragan A., Aguilar-Ferrandiz M.E., Correa-Rodriguez M. (2020). Assessing health science students’ health literacy and its association with health behaviours. Health Soc. Care Community.

[24] Edelmann D., Pfirrmann D., Heller S., Dietz P., Reichel J.L., Werner A.M., Schäfer M., Tibubos A.N., Deci N., Letzel S. (2022). Physical Activity and Sedentary Behavior in University Students–The Role of Gender, Age, Field of Study, Targeted Degree, and Study Semester. Front. Public Health.

[25] Chusak C., Tangmongkhonsuk M., Sudjapokinon J., Adisakwattana S. (2022). The Association between Online Learning and Food Consumption and Lifestyle Behaviors and Quality of Life in Terms of Mental Health of Undergraduate Students during COVID-19 Restrictions. Nutrients.

[26] Yamane T. (1973). Statistics, an Introductory Analysis.

[27] Ivanec P.T. (2022). The Lack of Academic Social Interactions and Students&rsquo; Learning Difficulties during COVID-19 Faculty Lockdowns in Croatia: The Mediating Role of the Perceived Sense of Life Disruption Caused by the Pandemic and the Adjustment to Online Studying. Soc. Sci..

[28] Maleki B. (2022). The Causal Model of Self-regulation in University Students based on Reducing Perceived Social Interactions during COVID-19: The Mediating Role of Adjustment to Online Learning. Int. J. Behav. Sci..

[29] Dhawan S. (2020). Online Learning: A Panacea in the Time of COVID-19 Crisis. J. Educ. Technol. Syst..

[30] Pardhan S., Parkin J., Trott M., Driscoll R. (2022). Risks of Digital Screen Time and Recommendations for Mitigating Adverse Outcomes in Children and Adolescents. J. Sch. Health.

[31] Tabares-Tabares M., Moreno Aznar L.A., Aguilera-Cervantes V.G., León-Landa E., López-Espinoza A. (2022). Screen use during food consumption: Does it cause increased food intake? A systematic review. Appetite.

[32] Hammoudi S.F., Mreydem H.W., Ali B.T.A., Saleh N.O., Chung S., Hallit S., Salameh P. (2021). Smartphone Screen Time Among University Students in Lebanon and Its Association with Insomnia, Bedtime Procrastination, and Body Mass Index during the COVID-19 Pandemic: A Cross-Sectional Study. Psychiatry Investig..

[33] Julian V., Haschke F., Fearnbach N., Gomahr J., Pixner T., Furthner D., Weghuber D., Thivel D. (2022). Effects of Movement Behaviors on Overall Health and Appetite Control: Current Evidence and Perspectives in Children and Adolescents. Curr. Obes. Rep..

[34] World Health Organization (2010). Global Recommendations on Physical Activity for Health.

[35] Arias-Palencia N.M., Solera-Martinez M., Gracia-Marco L., Silva P., Martinez-Vizcaino V., Canete-Garcia-Prieto J., Sanchez-Lopez M. (2015). Levels and Patterns of Objectively Assessed Physical Activity and Compliance with Different Public Health Guidelines in University Students. PLoS ONE.

[36] Gallo L.A., Gallo T.F., Young S.L., Moritz K.M., Akison L.K. (2020). The Impact of Isolation Measures Due to COVID-19 on Energy Intake and Physical Activity Levels in Australian University Students. Nutrients.

[37] Jia P., Zhang L., Yu W., Yu B., Liu M., Zhang D., Yang S. (2021). Impact of COVID-19 lockdown on activity patterns and weight status among youths in China: The COVID-19 Impact on Lifestyle Change Survey (COINLICS). Int. J. Obes..

[38] Katewongsa P., Widyastaria D.A., Saonuam P., Haematulin N., Wongsingha N. (2021). The effects of the COVID-19 pandemic on the physical activity of the Thai population: Evidence from Thailand’s Surveillance on Physical Activity 2020. J. Sport Health Sci..

[39] Papaconstantinou E., Quick V., Vogel E., Coffey S., Miller A., Zitzelsberger H. (2020). Exploring Relationships of Sleep Duration with Eating and Physical Activity Behaviors among Canadian University Students. Clocks Sleep.

[40] Al Khatib H.K., Harding S.V., Darzi J., Pot G.K. (2017). The effects of partial sleep deprivation on energy balance: A systematic review and meta-analysis. Eur. J. Clin. Nutr..

[41] Watson N.F., Badr M.S., Belenky G., Bliwise D.L., Buxton O.M., Buysse D., Dinges D.F., Gangwisch J., Grandner M.A., Kushida C. (2015). Recommended Amount of Sleep for a Healthy Adult: A Joint Consensus Statement of the American Academy of Sleep Medicine and Sleep Research Society. Sleep.

[42] Pengpid S., Peltzer K. (2020). Skipping Breakfast and Its Association with Health Risk Behaviour and Mental Health among University Students in 28 Countries. Diabetes Metab. Syndr. Obes. Targets Ther..

[43] Rehman R., Zafar A., Mohib A., Hussain M., Ali R. (2018). Self-reported academic performance in relation to health behaviours among Bahria University students. J. Pak. Med. Assoc..

[44] Adolphus K., Lawton C.L., Dye L. (2013). The effects of breakfast on behavior and academic performance in children and adolescents. Front. Hum. Neurosci..

[45] Pendergast F.J., Livingstone K.M., Worsley A., McNaughton S.A. (2016). Correlates of meal skipping in young adults: A systematic review. Int. J. Behav. Nutr. Phys. Act..

[46] Reuter P.R., Forster B.L., Brister S.R. (2021). The influence of eating habits on the academic performance of university students. J. Am. Coll. Health.

[47] Food and Agriculture Organization, International Fund for Agricultural Development, United Nations Children’s Fund, World Food Programme, World Health Organization (2020). The State of Food Security and Nutrition in the World 2020: Transforming Food Systems for Affordable Healthy Diets.

[48] van Vliet J.S., Gustafsson P.A., Nelson N. (2016). Feeling ‘too fat’ rather than being ‘too fat’ increases unhealthy eating habits among adolescents—Even in boys. Food Nutr. Res..

[49] Medin A.C., Myhre J.B., Diep L.M., Andersen L.F. (2019). Diet quality on days without breakfast or lunch—Identifying targets to improve adolescents’ diet. Appetite.

[50] Rodrigues P.R.M., Luiz R.R., Monteiro L.S., Ferreira M.G., Gonçalves-Silva R.M.V., Pereira R.A. (2017). Adolescents’ unhealthy eating habits are associated with meal skipping. Nutrition.

[51] Musaiger A.O., Awadhalla M.S., Al-Mannai M., AlSawad M., Asokan G.V. (2017). Dietary habits and sedentary behaviors among health science university students in Bahrain. Int. J. Adolesc. Med. Health.

[52] Pourmohammadi B., Jalilvand M.A. (2019). Prevalence of alcohol consumption and related factors among students of higher education centers in one of the northeastern cities of Iran. AIMS Public Health.

[53] Vidal E.J., Alvarez D., Martinez-Velarde D., Vidal-Damas L., Yuncar-Rojas K.A., Julca-Malca A., Bernabe-Ortiz A. (2018). Perceived stress and high fat intake: A study in a sample of undergraduate students. PLoS ONE.

[54] Yahia N., Brown C.A., Rapley M., Chung M. (2016). Level of nutrition knowledge and its association with fat consumption among college students. BMC Public Health.

[55] Mwafi N.R., Al-Rawashdeh I.M., Al-Kubaisy W.A., Ezzat W.R., Al-Qazaqi R.A., Salameh M.H. (2021). Prevalence and factors related to obesity and fast food consumption among Mutah University students, Jordan. J. Pak. Med. Assoc..

[56] Sahasakul Y., Aursalung A., Thangsiri S., Wongchang P., Sangkasa-Ad P., Wongpia A., Polpanit A., Inthachat W., Temviriyanukul P., Suttisansanee U. (2022). Nutritional Compositions, Phenolic Contents, and Antioxidant Potentials of Ten Original Lineage Beans in Thailand. Foods.

[57] Guasch-Ferré M., Liu X., Malik V.S., Sun Q., Willett W.C., Manson J.E., Rexrode K.M., Li Y., Hu F.B., Bhupathiraju S.N. (2017). Nut Consumption and Risk of Cardiovascular Disease. J. Am. Coll. Cardiol..

[58] Wallace T.C., Bailey R.L., Blumberg J.B., Burton-Freeman B., Chen C.O., Crowe-White K.M., Drewnowski A., Hooshmand S., Johnson E., Lewis R. (2020). Fruits, vegetables, and health: A comprehensive narrative, umbrella review of the science and recommendations for enhanced public policy to improve intake. Crit. Rev. Food Sci. Nutr..

