# Supplementary material for: Lifestyles, Food Consumption Frequencies, and Eating Behaviors among Three Main Disciplines of Undergraduate Students during the Early COVID-19 Outbreak in Thailand
Source: Nutrients. 2023 Jun 16;15(12):2765. doi: 10.3390/nu15122765 (PMC10304545; doi:10.3390/nu15122765)
Supplement: Supplementary file 1 [file nutrients-15-02765-s001.zip › nutrients-2358056-supplementary.pdf]

Supplementary Materials

Figure S1. BMI Distribution among Undergraduate Students by Disciplines of Study

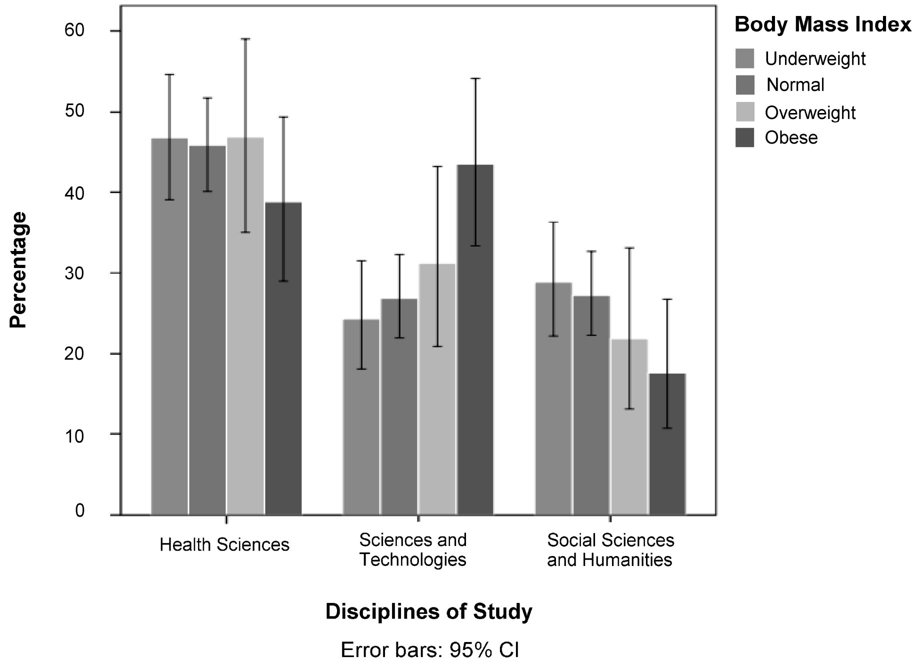

Figure S2. Association of BMI and Lifestyles (Hours per Week of Online Studying, Hours per Day of Social Media) among the Three Disciplines of Study

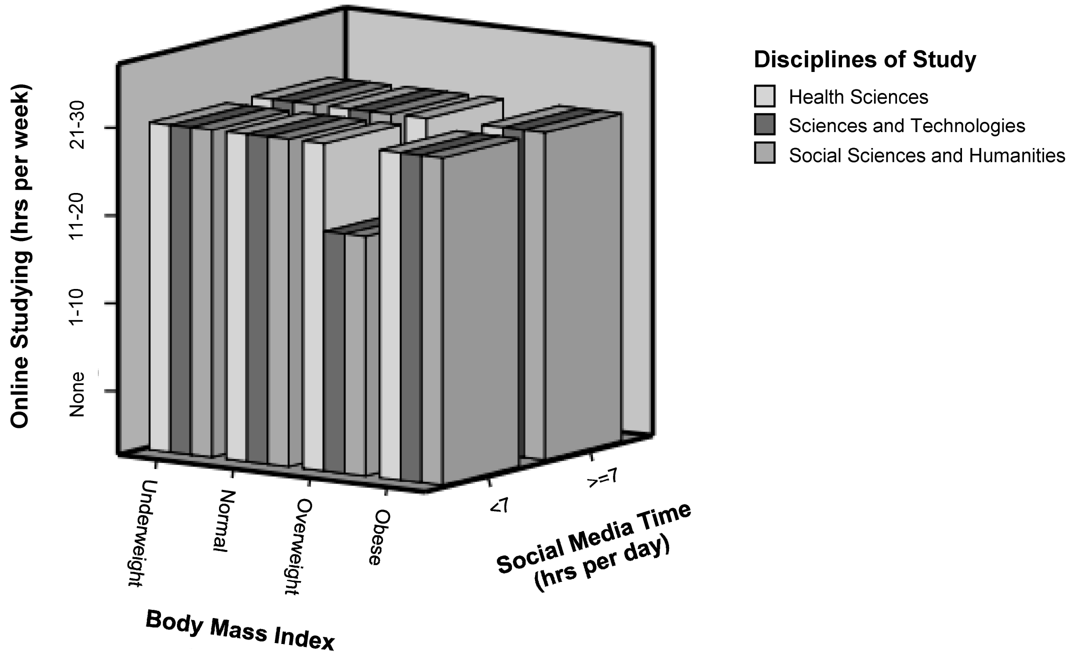

Figure S3. Association of BMI, Food Consumption Frequencies, and Lifestyle (Screen Time per Day) among the Three Disciplines of Study

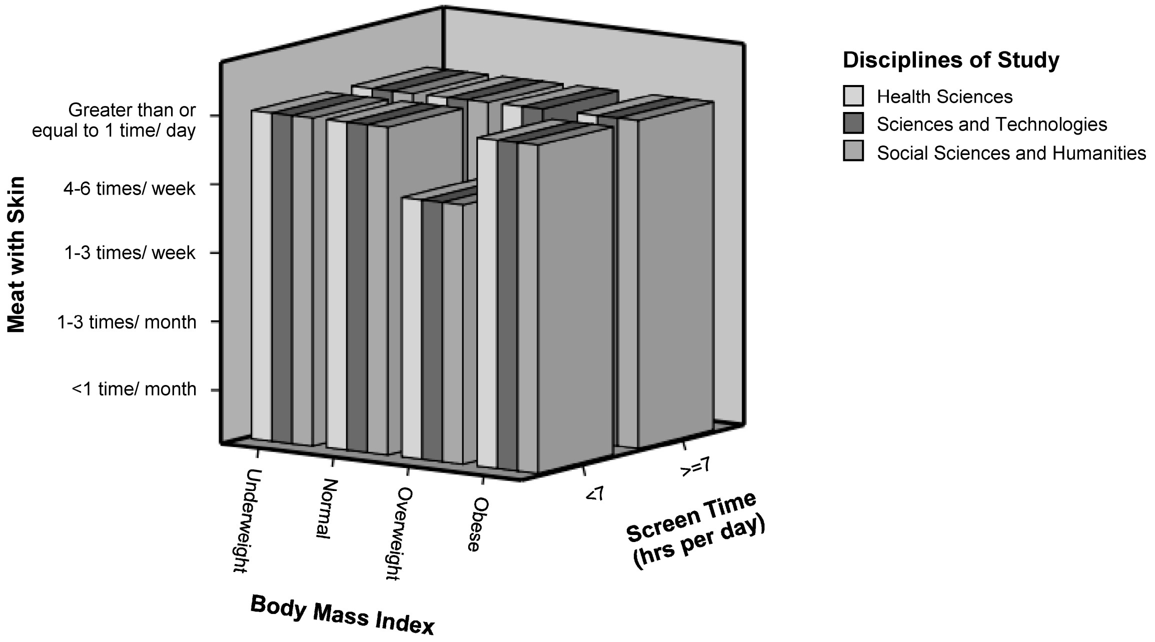

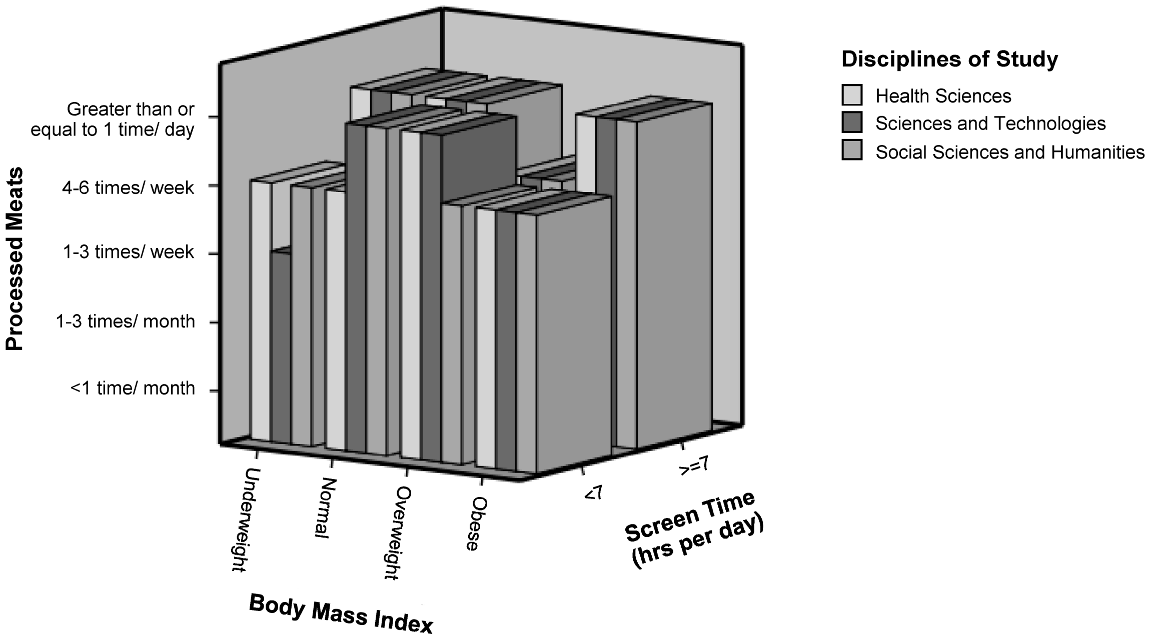

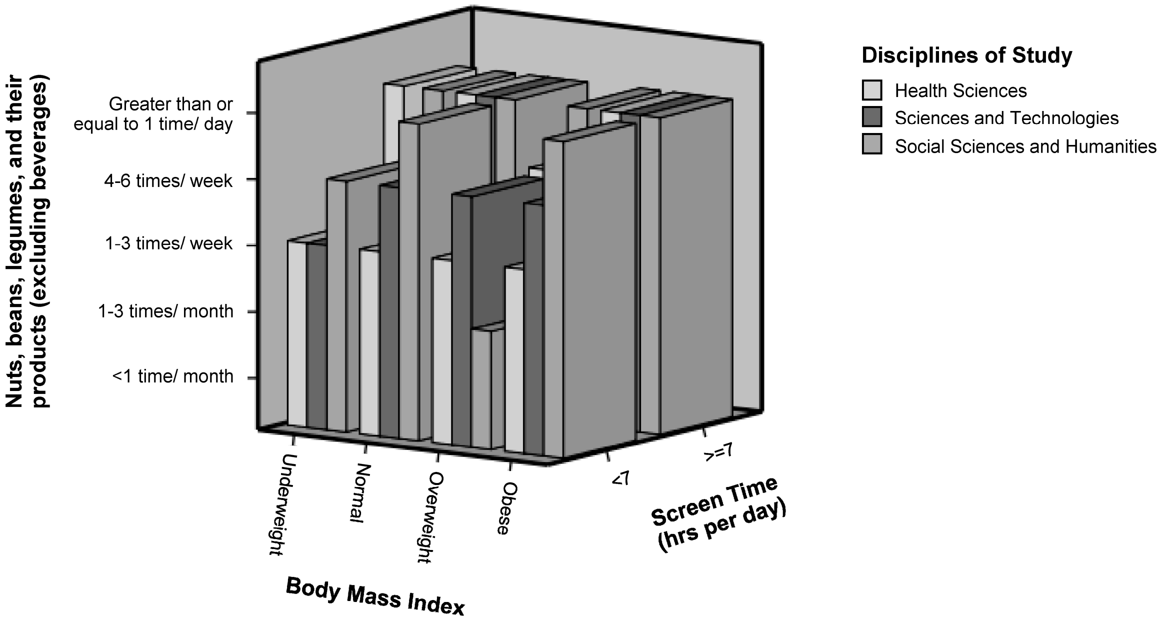
Nuts, beans, legumes, and their products (excluding beverages)
Greater than or equal to 1 time/ day
4-6 times/ week
1-3 times/ week
1-3 times/ month
<1 time/ month
Underweight
Normal
Overweight
Obese
Body Mass Index
<7
>=7
Screen Time (hrs per day)
Disciplines of Study
Health Sciences
Sciences and Technologies
Social Sciences and Humanities

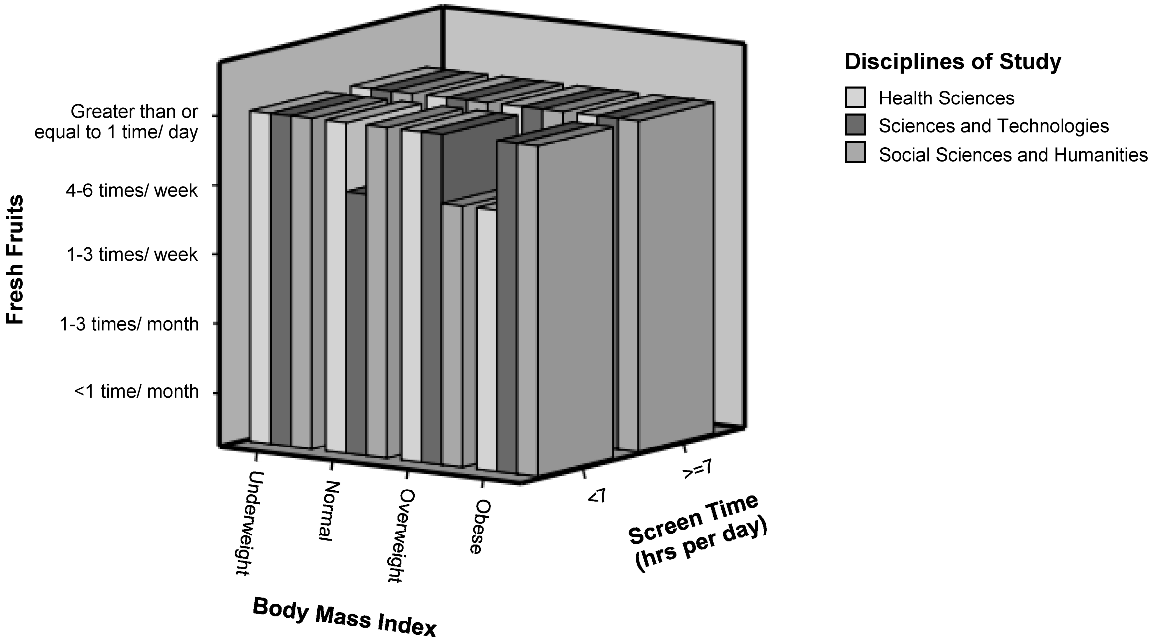
Fresh Fruits
Greater than or equal to 1 time/ day
4-6 times/ week
1-3 times/ week
1-3 times/ month
<1 time/ month
Underweight
Normal
Overweight
Obese
Body Mass Index
<7
>=7
Screen Time (hrs per day)
Disciplines of Study
Health Sciences
Sciences and Technologies
Social Sciences and Humanities

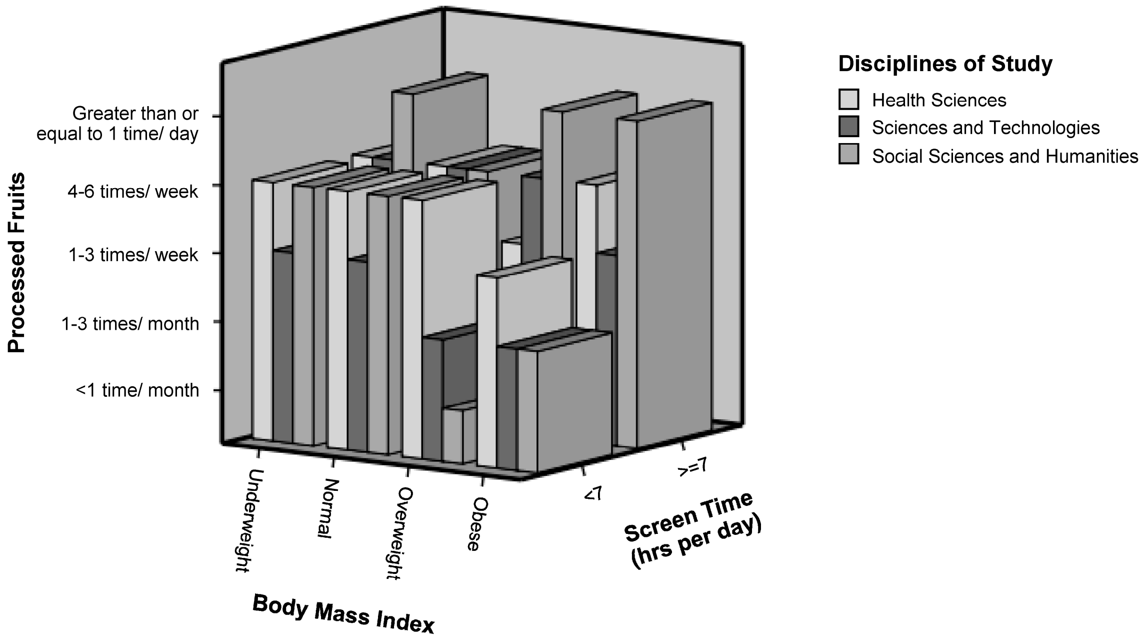
Disciplines of Study
Health Sciences
Sciences and Technologies
Social Sciences and Humanities
Processed Fruits
Greater than or equal to 1 time/ day
4-6 times/ week
1-3 times/ week
1-3 times/ month
<1 time/ month
Underweight
Normal
Overweight
Obese
Body Mass Index
<7
>=7
Screen Time (hrs per day)

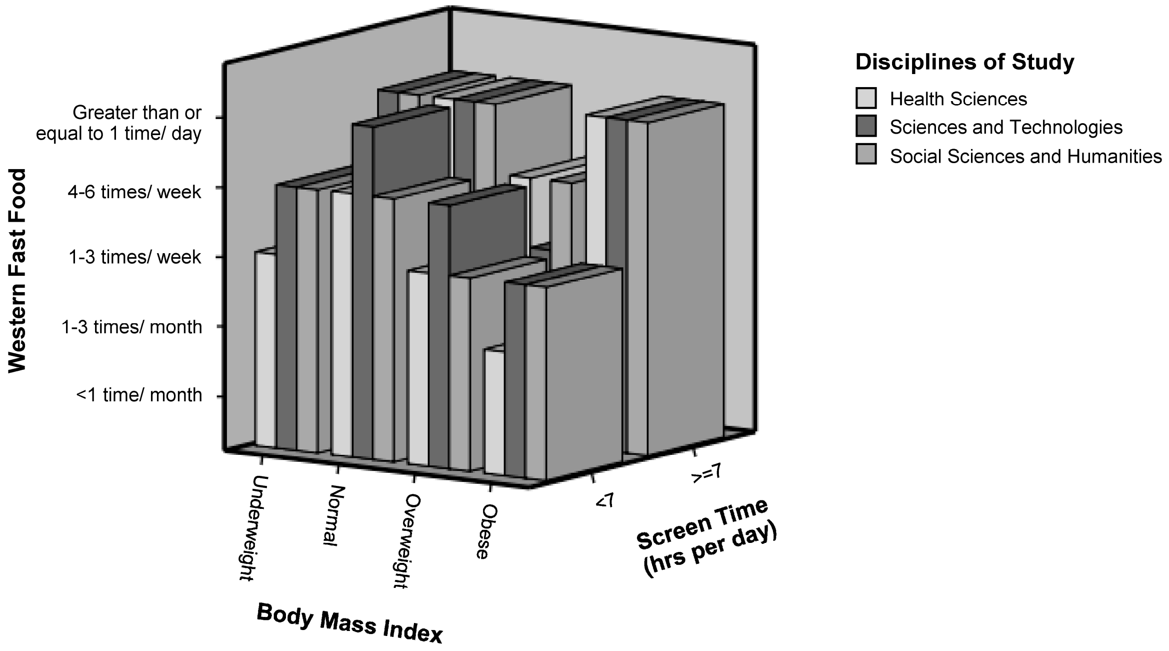
Disciplines of Study
Health Sciences
Sciences and Technologies
Social Sciences and Humanities
Western Fast Food
Greater than or equal to 1 time/ day
4-6 times/ week
1-3 times/ week
1-3 times/ month
<1 time/ month
Underweight
Normal
Overweight
Obese
Body Mass Index
<7
>=7
Screen Time (hrs per day)

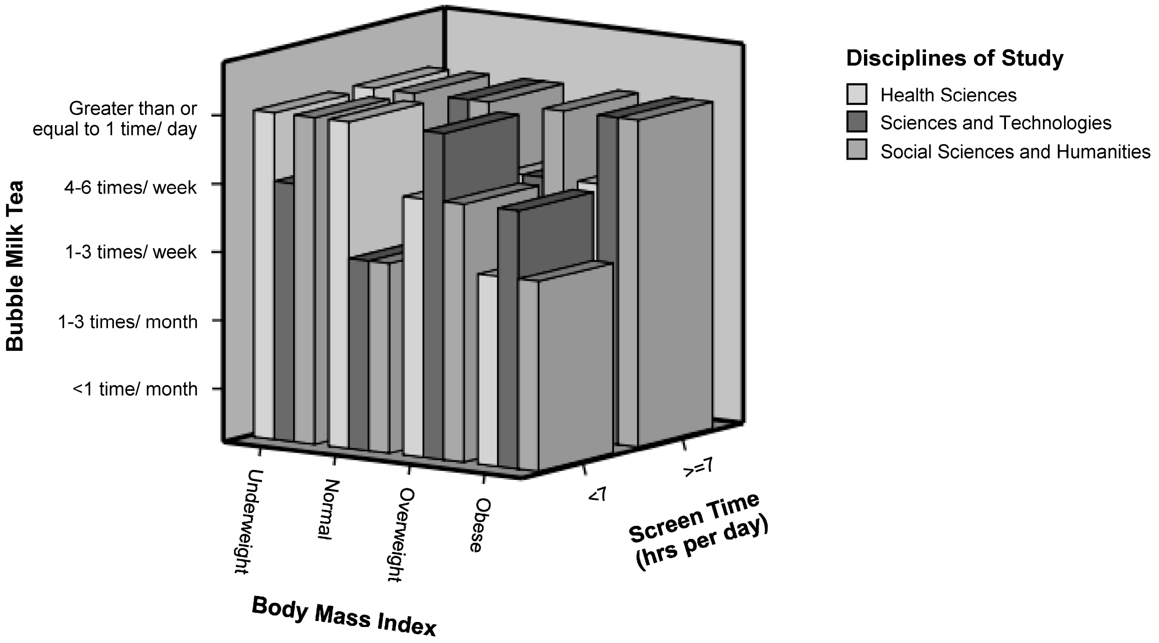
Bubble Milk Tea
Greater than or equal to 1 time/ day
4-6 times/ week
1-3 times/ week
1-3 times/ month
<1 time/ month
Underweight
Normal
Overweight
Obese
<7
>=7
Screen Time (hrs per day)
Body Mass Index
Disciplines of Study
Health Sciences
Sciences and Technologies
Social Sciences and Humanities

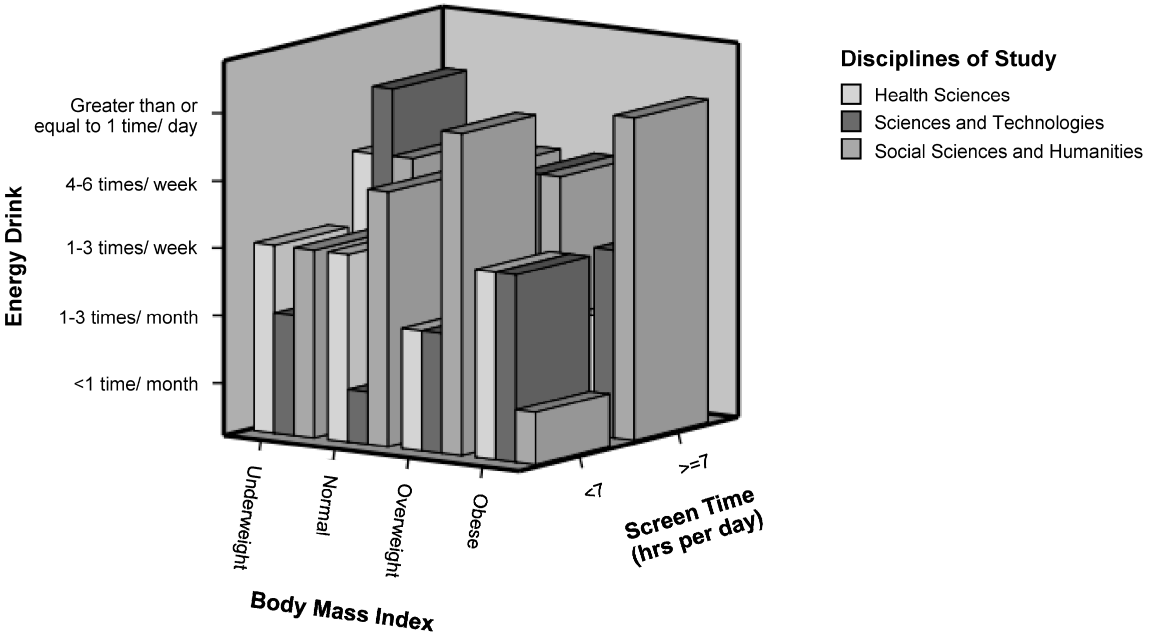
Energy Drink
Greater than or equal to 1 time/ day
4-6 times/ week
1-3 times/ week
1-3 times/ month
<1 time/ month
Underweight
Normal
Overweight
Obese
<7
>=7
Screen Time (hrs per day)
Body Mass Index
Disciplines of Study
Health Sciences
Sciences and Technologies
Social Sciences and Humanities

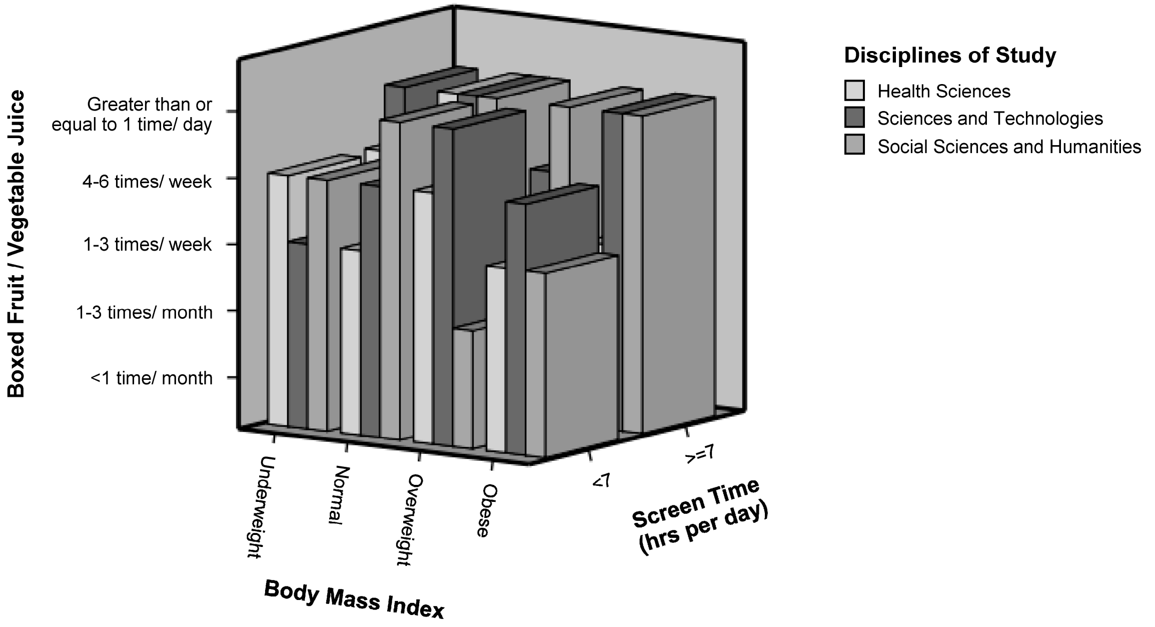
Disciplines of Study
Health Sciences
Sciences and Technologies
Social Sciences and Humanities
Boxed Fruit / Vegetable Juice
Greater than or equal to 1 time/ day
4-6 times/ week
1-3 times/ week
1-3 times/ month
<1 time/ month
Underweight
Normal
Overweight
Obese
<7
>=7
Screen Time (hrs per day)
Body Mass Index

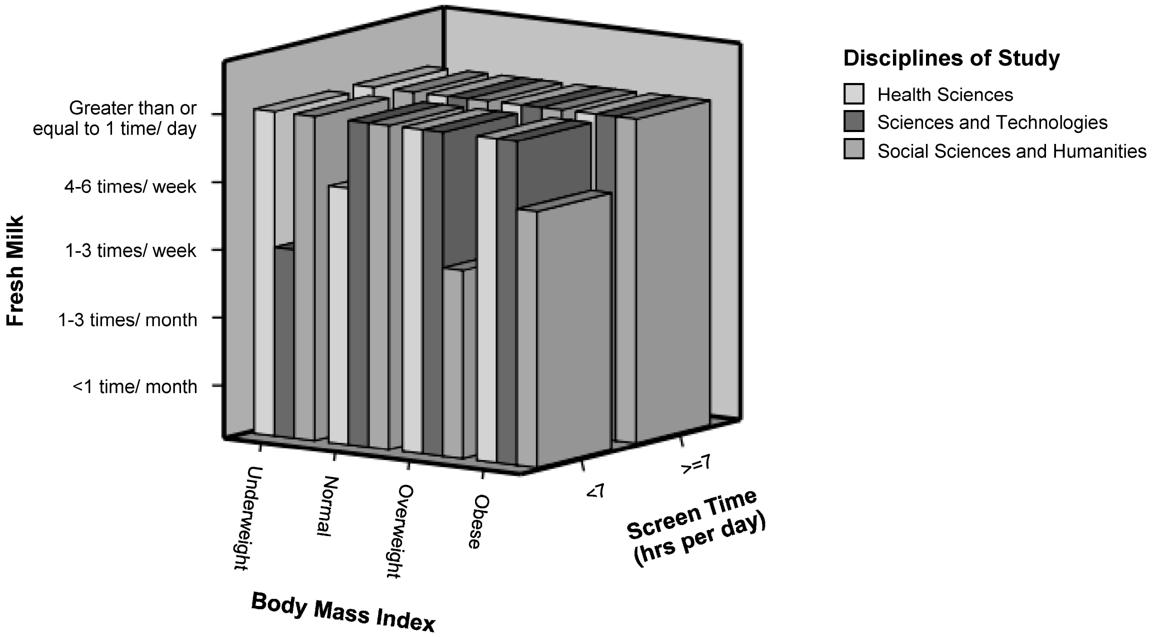
Disciplines of Study
Health Sciences
Sciences and Technologies
Social Sciences and Humanities
Fresh Milk
Greater than or equal to 1 time/ day
4-6 times/ week
1-3 times/ week
1-3 times/ month
<1 time/ month
Underweight
Normal
Overweight
Obese
<7
>=7
Screen Time (hrs per day)
Body Mass Index

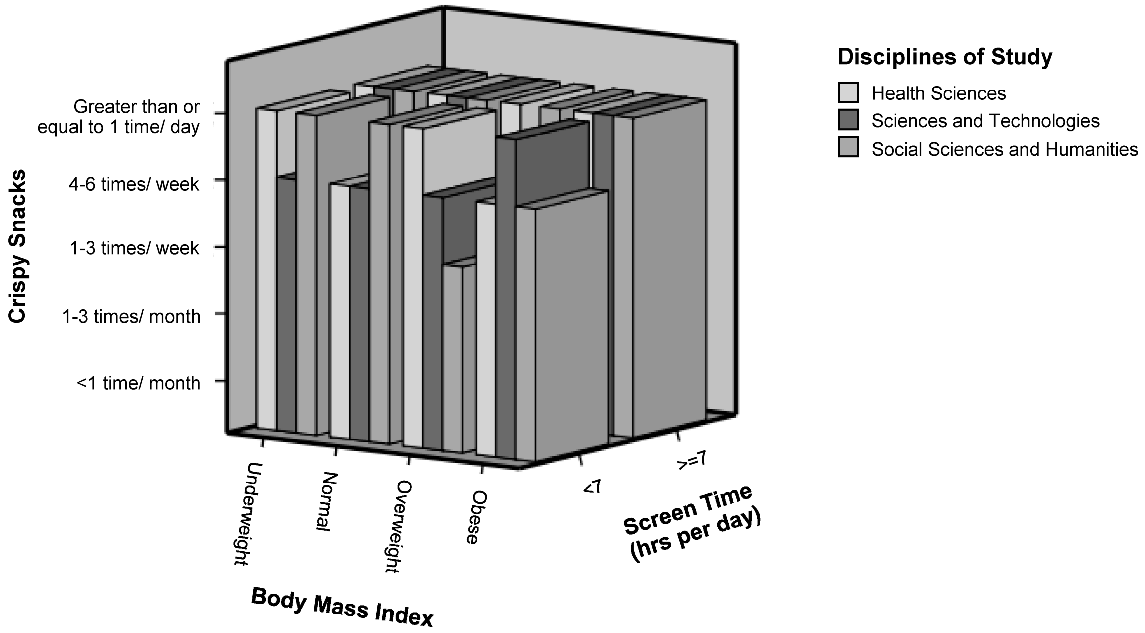

Crispy Snacks
Greater than or equal to 1 time/ day
4-6 times/ week
1-3 times/ week
1-3 times/ month
<1 time/ month
Underweight
Normal
Overweight
Obese
Body Mass Index
<7
>=7
Screen Time (hrs per day)
Disciplines of Study
Health Sciences
Sciences and Technologies
Social Sciences and Humanities
